# Del immune V and microbiome restructuring in colorectal cancer surgery: a randomized double blind placebo controlled trial

**DOI:** 10.3389/fcimb.2026.1853373

**Published:** 2026-07-07

**Authors:** Gissel García, Antonio Díaz, LLipsy T. Fernández, Mirka Bernal, Josanne Soto, Gilda Núñez, Vilma Fleites, Ernesto Arteaga, Liubov Sichel, Raúl de Jesús Cano

**Affiliations:** 1 Gissel García, Pathology Department, Hermanos Ameijeiras Clinical and Surgical Hospital, La Habana, Cuba; 2 Antonio Díaz.Statistical and Research Department, Hermanos Ameijeiras Clinical and Surgical Hospital, La Habana, Cuba; 3 Lllipsy T Fernández Surgery Department, Hermanos Ameijeiras Clinical and Surgical Hospital, La Habana, Cuba; 4 Mirka Bernal Clinical Laboratory Department, Hermanos Ameijeiras Clinical and Surgical Hospital, La Habana, Cuba; 5 Josanne Soto Clinical Laboratory Department, Hermanos Ameijeiras Clinical and Surgical Hospital, La Habana, Cuba; 6 Gilda Núñez Surgery Department, Hermanos Ameijeiras Clinical and Surgical Hospital, La Habana, Cuba; 7 Vilma Fleites: Oncology Department, Hermanos Ameijeiras Clinical and Surgical Hospital, La Habana, Cuba; 8 Ernesto Arteaga Pathology Department, Hermanos Ameijeiras Clinical and Surgical Hospital, La Habana, Cuba; 9 Liubov Sichel: Stellar Biotics, LLC., Rockleigh, NJ, United States; 10 Raúl de Jesús Cano, Biological Sciences Department, California Polytechnic State University, San Luis Obispo, CA, United States; 11 8 Academia de Ciencias de Cuba, La Habana, Cuba

**Keywords:** colorectal cancer surgery, del-immune V, gut microbiome, immunomodulation, inflammatory biomarkers, metabiotic, randomized double-blind trial, short-chain fatty acids

## Abstract

**Introduction:**

The gut microbiome is increasingly recognized as a central factor in carcinogenesis. Dietary components and therapeutic interventions, including probiotics, may influence microbial composition and function, thereby modulating cancer risk. Del−Immune V, a metabiotic supplement derived from Lactobacillus rhamnosus, has demonstrated immunomodulatory properties. This study investigates its role in microbiome restructuring and patient−reported outcomes in colorectal cancer patients during the perioperative period.

**Materials and Methods:**

A randomized, controlled, double−blind Phase I trial was conducted in 39 colorectal cancer patients undergoing elective resection, assigned to Del−Immune V (n=22) or placebo (n=17). Participants received two capsules daily (100 mg each), starting 7–15 days before surgery and continuing until 15 days postoperatively. Blood and fecal samples were collected at baseline and day 60 to assess IL−6, CRP, CEA, and microbiome composition. Patient−reported outcomes were measured using the EORTC QLQ−C30 questionnaire. Microbiome profiling was performed using 16S rRNA gene sequencing with PICRUSt-based functional inference.

**Results:**

Del−Immune V significantly reduced IL−6 (p=0.012) and supported CRP decline, while quality−of−life scores improved across multiple domains. Microbiome analyses revealed enrichment of short−chain fatty acid–producing genera (*Bifidobacterium, Agathobacter, Gemmiger, Phocaeicola*) and decline of CRC−associated taxa (*Fusobacterium*), with a significant improvement in the dysbiosis index (p=0.024).

**Conclusions:**

Del−Immune V demonstrated immunomodulatory activity, evidenced by reductions in IL−6 and CRP, alongside improvements in patient−reported quality of life. These effects were accompanied by restructuring of the gut microbiome, characterized by enrichment of protective commensals and reduction of CRC−associated taxa. Collectively, findings support Del−Immune V as a safe adjunctive therapy in colorectal cancer surgery, with potential to enhance recovery and long−term outcomes.

## 1 Introduction

The gut microbiome has emerged as a critical factor in the mechanisms of carcinogenesis, influencing tumor initiation, progression, and patient outcomes. Dietary components and therapeutic interventions, including probiotics, are increasingly studied for their ability to modulate microbial composition and function, thereby impacting cancer risk and recovery (Pidgorskyy et al., 2008; Mehta et al., 2023; Furnari et al., 2025; Allela et al., 2026). Del−Immune V is an FDA−registered nutritional supplement composed of lyophilized lysate of fermented cells of Lactobacillus rhamnosus V (DV strain), a Gram−positive bacterium. This metabiotic formulation contains cell wall fragments, including peptidoglycan−derived muramyl peptides and dipeptides, as well as essential nucleotides and amino acids, which act as microbe−associated molecular patterns (MAMPs) to stimulate innate immune cells through pattern−recognition receptors. Beyond its immunomodulatory activity, Del−Immune V may influence microbiome turnover, supporting microbial balance and reducing dysbiosis in vulnerable settings such as colorectal cancer surgery (Sichel et al., 2013; García Menéndez et al., 2024).

Colorectal cancer is the second leading cause of cancer in women and the third in men worldwide, with approximately half of patients developing metastatic disease (Siegel et al., 2024). Evidence indicates that an inflammatory microenvironment, closely linked to microbiome disruption, plays a decisive role in tumor progression (Xu et al., 2016; Cotan et al., 2024; Gwenzi et al., 2024). Inflammatory biomarkers such as interleukin−6 (IL−6) and C−reactive protein (CRP) are widely used to monitor this environment and guide therapeutic decisions (Xu et al., 2016; Cotan et al., 2024; Gwenzi et al., 2024).

During the perioperative period, the intestinal microbiota is particularly vulnerable to surgical trauma, intestinal cleansing, prophylactic antibiotics, hypoxia, and nutrient deprivation, all of which contribute to dysbiosis and increase the risk of complications (Cong et al., 2018; Koliarakis et al., 2020). Consequently, strategies to preserve or restore microbial balance are of growing interest, including prebiotics, probiotics, and symbiotics (Pitsillides et al., 2021; Cronin et al., 2022; Tweed et al., 2022).

Digestive surgeons have increasingly sought approaches to minimize dysbiosis. Preoperatively, colon preparation is avoided, and prophylactic antibiotic use is redefined due to their detrimental impact on microbiota. Intraoperatively, refined surgical techniques aim to reduce bleeding, transfusions, and tissue trauma, while favoring minimally invasive approaches with lower inflammatory impact (Tweed et al., 2022). Beyond surgical technique, interest has expanded toward microbiome modulation through dietary and probiotic interventions, reflecting the exponential growth in their clinical use (Pitsillides et al., 2021).

All patients in this study were managed under the Enhanced Recovery After Surgery (ERAS) protocol, which integrates preoperative, intraoperative, and postoperative strategies to minimize surgical stress, accelerate recovery, and improve outcomes in colorectal surgery (Holder-Murray et al., 2019; Zhang et al., 2019; Smith et al., 2020; Wei et al., 2020). Key elements include nutritional optimization, fluid balance, multimodal pain control, early enteral nutrition, and mobilization. Adherence to ERAS principles has been shown to reduce morbidity, shorten hospital stay, and improve long−term outcomes (Zhang et al., 2019; Wei et al., 2020). Within this framework, modulation of the microbiome through probiotic interventions such as Del−Immune V may represent a novel adjunctive approach to reduce systemic inflammation, improve microbial balance, and enhance quality of life.

We hypothesize that reductions in inflammatory biomarkers such as CRP and IL−6 will serve as primary outcome measures, while microbiome restructuring will provide mechanistic insights into how probiotics influence carcinogenesis. The primary outcome of this work is to evaluate the effects of Del−Immune V on microbiome turnover and quality of life in colorectal cancer patients during the peri− and postoperative period. The secondary outcome is to analyze specific microbiome changes induced by Del−Immune V.

## 2 Materials and methods

### 2.1 Study design

This proof-of-concept, randomized, controlled, double-blind Phase I clinical trial was conducted to evaluate the metabiotic compound Del-Immune V as a complementary perioperative therapy in patients with colorectal cancer at any stage of disease. The study was designed not only to assess clinical outcomes but also to explore how probiotic-based modulation may influence microbiome restructuring, systemic inflammation, and patient-reported quality of life, recognizing the central role of the gut microbiome in carcinogenesis.

### 2.2 Ethical aspect considerations

This is a randomized, controlled, double-blind, Phase I study with the investigational product Del-Immune V, which has been registered as a nutritional supplement by the Regulatory Agency of the Cuban National Registry (Nutrition Institute of Food Hygiene (INHA), belonging to the National Institute of Hygiene and Epidemiology INHEM) of Cuba (Health License No. Pl-30490/22). The study protocol was reviewed and approved by the Ethics Committee of the Hermanos Ameijeiras Clinical and Surgical Hospital (approval number NA241LH001-23; May 11, 2022, modified September 15, 2024) and registered in the Cuban Public Registry of Clinical Trials with the code RPCEC00000414. The clinical trial was carried out in accordance with the provisions of the Declaration of Helsinki of the World Medical Assembly (World Medical Association, 2013), with the last update at the 64th General Assembly in Fortaleza, Brazil, October 2013, and with the current state regulations for nutritional supplements of the Institute of Food Hygiene and Nutrition (INHA). All participants read and signed an informed consent form prior to enrollment in the study.

### 2.3 Eligibility criteria

Eligible participants included adult patients (≥18 years), of any sex, residing in Cuba, who met diagnostic criteria requiring colon or rectal resection, had a Karnofsky Performance Status ≥70%, and provided written informed consent. Exclusion criteria comprised pregnancy or breastfeeding, emergency surgery, concurrent participation in another investigational study, presence of brain metastases, or mental disorders that could compromise adherence or data collection. Patients were withdrawn from the study if they underwent colostomy, experienced serious adverse events attributable to the supplement requiring treatment interruption, voluntarily abandoned participation, developed conditions deemed incompatible with continuation by the investigator, failed to adhere to supplement intake for more than 15 consecutive days, or died during the study. Importantly, all patients who discontinued treatment remained part of the study population and were included in the final analysis.

### 2.4 Statistical methodology and variable definition

The sample size was calculated based on a previous pilot study that demonstrated a 40% reduction in postoperative IL-6 levels with Del-Immune V. Assuming a statistical power of 80% and a significance level of α = 0.05, a minimum of 18 patients per group was estimated. Considering a potential dropout rate of 15%, the study planned to include 22 patients in the intervention group and 17 patients in the placebo group, for a total of 39 patients. This sample size was deemed sufficient for this Phase I proof-of-concept trial to detect meaningful changes in primary inflammatory biomarkers and generate preliminary mechanistic data on microbiome restructuring. Patients were then randomly assigned in a 1:1 ratio to receive Del−Immune V (Batch 426) or placebo (Batch 425) using a computer−generated randomization sequence. Both investigators and patients remained blinded to treatment allocation until completion of the statistical analysis.

The primary variables included IL−6 levels (pg/mL) at week 8 postoperatively and the percentage changes in IL−6 from baseline to week 8. Secondary variables comprised CRP (mg/L) and CEA (ng/mL) levels at week 8, perioperative complications (Yes/No), length of hospital stay (days), health−related quality of life assessed with the EORTC QLQ−C30 and reported adverse and beneficial effects. Safety variables encompassed the incidence of adverse effects categorized by severity (mild, moderate, severe), clinical laboratory parameters and vital signs, as well as treatment tolerance.

Regarding multiple testing, IL-6 was the pre-specified primary endpoint; therefore, no multiplicity adjustment was required for its primary analysis. For secondary and exploratory endpoints (e.g., CRP, CEA, and quality-of-life sub-scales), p-values are reported as nominal. In the con (Rothman, 1990)text of this Phase I proof-of-concept trial with a modest sample size (n=39), strict corrections (such as Bonferroni) for secondary clinical endpoints were intentionally avoided to prevent an inflated Type II error rate (false negatives), prioritizing instead the magnitude of effect and consistent directionality of the findings (Rothman, 1990; Feise, 2002). Conversely, for high-dimensional microbiome data (e.g., PICRUSt functional profiling), where the risk of false positives is substantial, we rigorously applied False Discovery Rate (FDR) correction (Benjamini-Hobberg method) to control for multiple comparisons (Benjamini and Hochberg, 1995).

### 2.5 Patient enrollment and analysis sets

**Figure 1:**
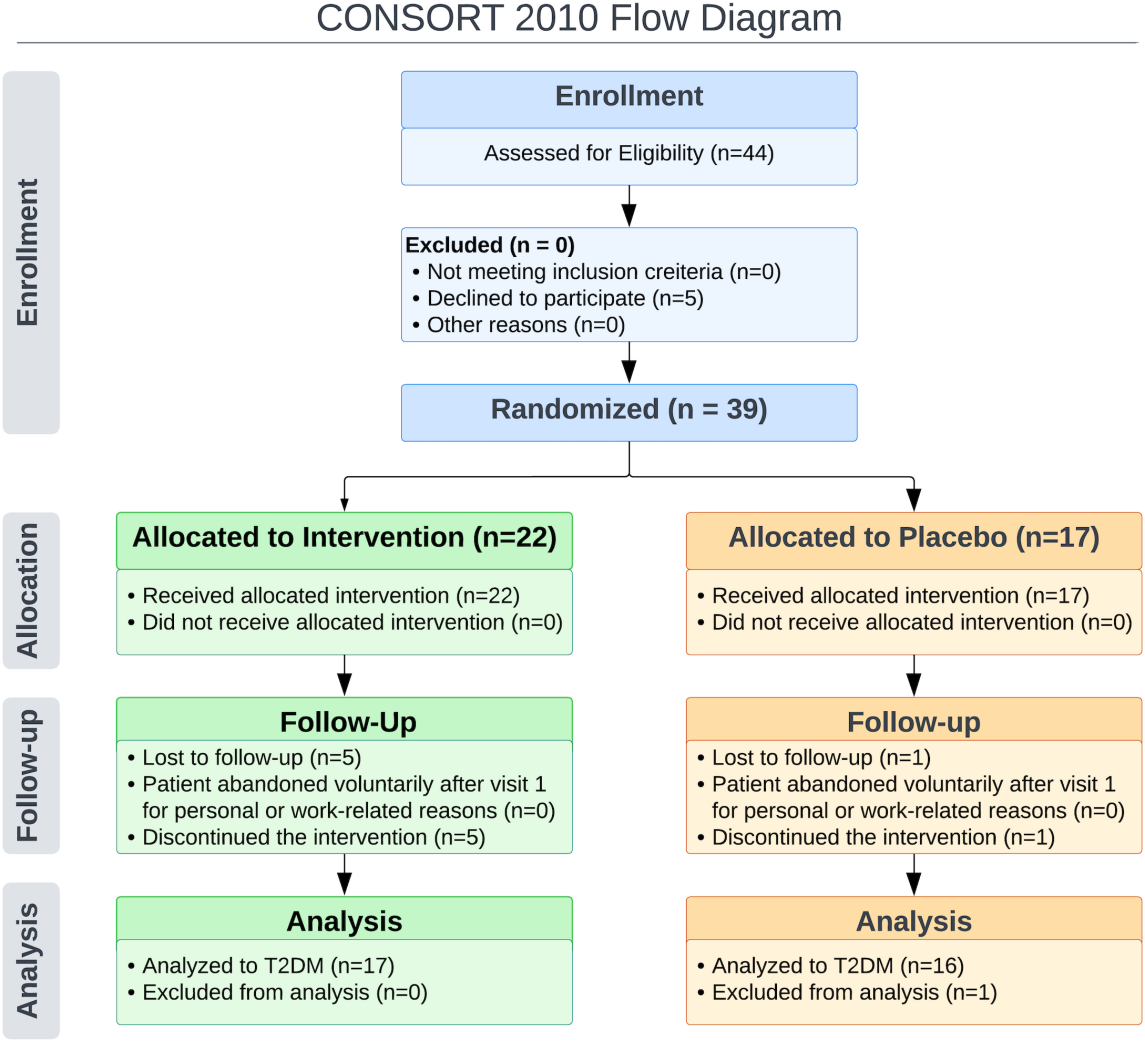
CONSORT flow diagram detailing patient recruitment, randomization, allocation, and follow-up. The diagram illustrates the progression of all enrolled patients (N = 44) through the trial phases, resulting in a final analyzed cohort of n = 39 patients (n = 22 in the Del-Immune V group and n = 17 in the Placebo group). Reasons for exclusion and dropout are specified for each stage **Figure 2**.

The intention-to-treat (ITT) principle was applied for all primary analyses. The per-protocol population included all patients who completed at least 80% of the assigned treatment and had complete evaluations at baseline and week 8. Participants were eligible if they were ≥18 years of age, of either sex, with clinical, endoscopic, and imaging-confirmed colorectal cancer requiring elective surgical resection, and who provided written informed consent. During the one-year recruitment period, however, a total of 44 patients were enrolled. Of these, 39 underwent elective colorectal cancer resection and were randomized in a 1:1 ratio into two study arms: Del-Immune V (n = 22) and Placebo (n = 17). This final distribution was considered sufficient to support the planned exploratory analyses, while acknowledging the limitation posed by the smaller-than-expected sample size. Participant flow throughout the study is illustrated in the CONSORT flow diagram (**Figure 1**).

### 2.6 Treatment

Participants were randomized into two study arms. Group A (Placebo) received two capsules daily, administered orally every 12 hours, starting 7 to 15 days prior to surgery and continued uninterruptedly throughout the perioperative period and for 15 days postoperatively. The placebo consisted of an inert compound of rice flour (3.53 g per capsule). Group B (Del-Immune V) received two capsules daily, administered in the same manner as Group A. Del-Immune V is a nutritional supplement containing dried lysate of fermented cells of *Lactobacillus rhamnosus* V.

Capsules were dispensed by a pharmacy specialist responsible for product storage and temperature control. Delivery occurred seven days before surgery, coinciding with the patient’s visit to the General Surgery clinic for admission scheduling. Each treatment package was provided in clearly labeled envelopes to ensure proper identification and adherence.

The dose of Del-Immune V administered in this study (100 mg twice daily; total 200 mg/day) was chosen according to the manufacturer’s recommended regimen for human use, which has been widely adopted in routine practice without reported safety concerns (Savytska et al., 2026). In addition, preclinical studies have demonstrated dose-dependent immunomodulatory activity of Del-Immune V. Notably (Sichel et al., 2013), reported that oral administration of 50 μg/mouse induced significant interferon production and moderate stimulation of TNF, identifying this dose as optimal for sustained interferonogenic activity *in vivo*. These findings provided biological support for the translation of the commercially recommended dose into human clinical evaluation (Sichel et al., 2013). Based on this evidence, our phase I trial focused on assessing tolerability and preliminary biological effects at the established safe dose, rather than exploring dose escalation.

### 2.7 Sample collection, processing, and data management

Sample collection, supplement delivery, and clinical evaluation were performed at baseline (week −1, prior to surgery) and at the end of the treatment period (day 60 post-surgery). Blood samples were obtained via venipuncture, labeled with the participant’s inclusion number, processed, and aliquoted within one hour for storage and subsequent analyses.

In addition, fecal samples were collected from a subset of 17 individuals (10 from the placebo group and 7 from the Del-Immune V group), both prior to treatment initiation and at the conclusion of the intervention, to evaluate changes in gut microbiome composition. Fecal specimens were obtained using standardized collection kits designed to preserve microbial nucleic acids and proteins at ambient temperature. Participants were instructed to follow the manufacturer’s guidelines for sample collection, and samples were stored at −20 °C until further processing.

Beyond biological samples, detailed clinical and pathological data were recorded for each patient, including tumor differentiation grade, tumor location, lymphovascular invasion, depth of infiltration, lymph node involvement, hospital stay duration, postoperative recovery, and whether reintervention was required. Information on oncological treatment and follow-up was also documented. Adverse and beneficial effects were monitored throughout the study.

To assess patient-reported outcomes, the EORTC QLQ-C30 questionnaire (Kaasa et al., 1995) was administered to all participants at baseline and at the end of treatment, providing standardized measures of health-related quality of life.

All participant records were maintained in a secure, dedicated database with restricted access limited to study and clinical staff. The Health and Hospital Administration (HHA) oversaw the security of the information technology infrastructure supporting data management.

### 2.8 Clinical determinations

For the evaluation of IL-6, carcinoembryonic antigen (CEA), and C-reactive protein (CRP) biomarkers, serum samples were analyzed using a Cobas 6000 modular immunochemical autoanalyzer (Roche Diagnostics, Basel, Switzerland), following the manufacturer’s recommended protocols.

### 2.9 Metagenomic taxonomic profiling

To characterize microbial community structure and functional potential, fecal samples were profiled using 16S rRNA gene sequencing combined with PICRUSt-based functional inference. This workflow achieved genus-level taxonomic resolution and inferred functional pathways from amplicon data. The following sections describe sequence processing, quality control, taxonomic classification, diversity analyses, and functional inference procedures.

#### 2.9.1 Sequence processing and quality control

For paired-end sequencing, reads representing each end of the same DNA fragment were merged using the VSEARCH program (Rognes et al., 2016) to obtain overlapping sequence information. Primers and adapter sequences were removed using an in-house script, followed by quality filtering to eliminate low-quality reads, chimeric sequences, sequencing artifacts, and reads not predicted to originate from 16S rRNA genes. The resulting high-quality sequences were queried against the EzBioCloud 16S database (Chalita et al., 2024) using VSEARCH to determine taxonomic similarity. After quality filtering and chimera removal, the mean sequencing depth was 68,273 ± 11,481 reads per sample.

A cutoff of 97% sequence similarity was applied for species-level identification. Sequences not matching a reference sequence at ≥97% similarity were clustered into Operational Taxonomic Units (OTUs) using UCLUST (Prasad et al., 2015; James et al., 2018) at a 97% similarity threshold. OTU abundance tables were subsequently used to calculate alpha-diversity metrics, including species richness, Shannon diversity, and Simpson diversity indices.

To account for differences in sequencing depth among samples and the compositional nature of microbiome data, abundance tables were normalized prior to downstream statistical analyses using centered log-ratio (CLR) transformation. Beta-diversity analyses, taxonomic comparisons, and functional pathway analyses were performed using normalized datasets.

#### 2.9.2 EzBioCloud platform

EzBioCloud (ChunLab, Inc.) integrates curated bacterial, archaeal, and fungal 16S rRNA databases for accurate classification. It provides alpha and beta diversity analyses (Shannon, Simpson, Bray−Curtis) and predictive tools such as PICRUSt for functional pathway inference. Biomarker discovery was performed using LEfSe (Linear Discriminant Analysis Effect Size) (Khleborodova et al., 2024).

#### 2.9.3 Application in the study

Fecal samples collected at baseline, day 60, and EOS were processed through EzBioCloud for taxonomic profiling, diversity analysis, and functional predictions. Outputs included identification of differentially abundant taxa, enrichment of SCFA−producing microbes, and reductions in inflammatory taxa. Visualization tools (heatmaps, PCoA plots, phylogenetic trees) highlighted microbial dynamics.

#### 2.9.4 Functional profiling and dysbiosis index

Functional annotations were inferred using PICRUSt (Phylogenetic Investigation of Communities by Reconstruction of Unobserved States) (Douglas et al., 2020), which predicts metagenome functional content based on 16S rRNA marker gene data and a reference genome database. KEGG (Kanehisa and Goto, 2000) orthologs were quantified and pathways inferred using MinPath to minimize false positives. Pathway abundance was normalized for sequencing depth, enabling comparisons of predicted metabolic functions across groups. Differential functional features between the Del-Immune V and placebo groups were identified using LEfSe (Linear Discriminant Analysis Effect Size) (Khleborodova et al., 2024), with an LDA score threshold of ≥2.0.

A dysbiosis index (DI) was calculated to quantify shifts in the gut microbial community toward a dysbiosis state. The index was defined as the logarithmic ratio between bacterial families generally associated with microbial homeostasis and those associated with dysbiotic or inflammatory gut environments.

Relative abundance values derived from 16S rRNA gene sequencing were aggregated at the family or genus level. The dysbiosis index was calculated using the following formula:


$$DI\;=\;Log_{10}(\frac{Lachnospiraceae\;+\;Bifidobacteriaceae}{Alistipes\;+\;Fusobacterium\;+\;Parvimonas\;+\;Peptostreptococcus})$$

To compare diversity across Baseline, Placebo, and Treated groups (day 84), we applied richness metrics (ACE (Elena Schmitz and Rahmann, 2025), Chao1 (Deng et al., 2024), observed OTUs (Chiarello et al., 2022)) and diversity indices (Shannon (Kitikidou et al., 2024), Simpson (Bollarapu et al., 2024)). Phylogenetic diversity was assessed using PD (Faith, 2007), which measures total branch length of observed species. All metrics were calculated on rarefied data to ensure consistent sequencing depth. Statistical analyses included ANOVA (Kim, 2017) or Kruskal-Wallis (Chan and Walmsley, 1997) tests, with post-hoc tests applied where appropriate (p<0.05).picrust.

## 3 Results

### 3.1 Baseline clinical and pathological characteristics

A total of 39 patients undergoing elective colorectal cancer resection were included in the final analysis, randomized into two groups: Del-Immune V (n = 22) and Placebo (n = 17). Baseline demographic, clinical, and biomarker characteristics are summarized in **Table 1**.

**Table 1:** Baseline demographic, clinical, and biomarker characteristics of patients.

Variable	Metric	Del-immune V (n=22)	Placebo (n=17)	P-value
DEMOGRAPHIC DATA	Age (years), median (IQR)	68.0 (60.0-75.0)	66.0 (59.0-78.0)	0.891^1^
	Female sex, n (%)	14 (63.6%)	12 (70.6%)	0.736^2^
	Male sex, n (%)	8 (36.4%)	5 (29.4%)	
	White skin color, n (%)	17 (77.3%)	14 (82.4%)	0.709^2^
	Non-white skin color, n (%)	5 (22.7%)	3 (17.6%)	
	Height (m), mean ± SD	1.63 ± 0.11	1.61 ± 0.09	0.512^3^
	Baseline weight (kg), mean ± SD	66.8 ± 13.2	64.3 ± 15.1	0.578^3^
	Baseline BMI (kg/m²), median (IQR)	24.6 (22.6-27.7)	25.8 (20.8-28.7)	0.867^1^
PERSONAL MEDICAL HISTORY	Arterial hypertension, n (%)	8 (36.4%)	7 (41.2%)	0.759^2^
	Type 2 diabetes mellitus (T2DM), n (%)	2 (9.1%)	1 (5.9%)	1.000^2^
	Heart disease, n (%)	2 (9.1%)	0 (0%)	0.487^2^
	Prior or familial cancer, n (%)	11 (50.0%)	9 (52.9%)	0.853^2^
	No comorbidities, n (%)	7 (31.8%)	5 (29.4%)	1.000^2^
BASELINE BIOMARKERS (Week 0)	IL-6 (pg/mL), median (IQR)	8.71 (3.33-22.43)	11.58 (4.23-39.8)	0.456^1^
	CRP (mg/L), median (IQR)	12.82 (5.37-48.12)	15.75 (3.70-40.28)	0.734^1^
	CEA (ng/mL), mediana (RIC)	4.77 (2.01-8.88)	3.16 (1.78-8.87)	0.357^1^
	Elevated IL-6 (>5 pg/mL), n (%)	15 (68.2%)	13 (76.5%)	0.736^2^
	Elevated CRP (>5 mg/L), n (%)	17 (77.3%)	13 (76.5%)	1.000^2^
	Elevated CEA (>4.7 ng/mL), n (%)	11 (50.0%)	7 (41.2%)	0.588^2^
TUMOR CHARACTERISTICS	Moderately differentiated grade, n (%)	19 (86.4%)	15 (88.2%)	0.712^2^
	Right colon location, n (%)	11 (50.0%)	8 (47.1%)	0.956^2^
	Left colon location, n (%)	7 (31.8%)	6 (35.3%)	
	Lymphovascular invasion, n (%)	10 (45.5%)	7 (41.2%)	0.789^2^
	Metastatic lymph nodes (N+), n (%)	14 (63.6%)	10 (58.8%)	0.759^2^
TNM STAGING, n (%)	Stage I	1 (4.5%)	0 (0%)	0.056^2^
	Stage II	4 (18.2%)	4 (23.5%)	
	Stage III	12 (54.5%)	11 (64.7%)	
	Stage IV	5 (22.7%)	0 (0%)	
SURGICAL DATA	ERAS protocol, n (%)	22 (100%)	17 (100%)	1.000^2^
	Hospital stay (days), median (IQR)	5.0 (3.0-7.0)	3.0 (3.0-5.0)	0.070^1^
	Reintervention, n (%)	4 (18.2%)	0 (0%)	0.056^2^
	30-day mortality, n (%)	1 (4.5%)	0 (0%)	1.000^2^
ONCOLOGICAL TREATMENT, n (%)	Adjuvant chemotherapy	15 (68.2%)	13 (76.5%)	0.736^2^
	FOLFOX	10 (45.5%)	8 (47.1%)	0.923^2^
	XELOX	5 (22.7%)	5 (29.4%)	0.736^2^
	No chemotherapy	7 (31.8%)	4 (23.5%)	0.557^2^

*Data are presented as median (interquartile range, IQR), mean ± standard deviation (SD), or frequency (percentage), as appropriate. Statistical comparisons between groups were performed using the Mann–Whitney U test (¹), Fisher’s exact test (²), or Student’s t-test (³). BMI, body mass index; IL-6, interleukin-6; CRP, C-reactive protein; CEA, carcinoembryonic antigen; ERAS, Enhanced Recovery After Surgery; TNM, tumor-node-metastasis; SD, standard deviation; IQR, interquartile range.

### 3.2 Demographic data

The median age was 68.0 years (IQR: 60.0–75.0) in the Del-Immune V group and 66.0 years (IQR: 59.0–78.0) in the placebo group (p = 0.891). No significant differences were observed in sex distribution (female: 63.6% vs. 70.6%, p = 0.736), skin color (white: 77.3% vs. 82.4%, p = 0.709), or baseline body mass index (24.6 vs. 25.8 kg/m², p = 0.867). These findings confirm that the two groups were comparable with respect to fundamental demographic characteristics.

### 3.3 Personal medical history

The prevalence of arterial hypertension was similar between groups (36.4% vs. 41.2%, p = 0.759). Type 2 diabetes mellitus (T2DM) was present in 9.1% of the Del-Immune V group versus 5.9% of the Placebo group (p = 1.000). The history of prior or familial cancer was evenly distributed (50.0% vs. 52.9%, p = 0.853). Overall, no significant differences were observed in comorbidity burden between groups (all p > 0.05).

### 3.4 Baseline inflammatory and oncological biomarkers

Baseline serum levels of interleukin-6 (IL-6) were comparable between groups [8.71 pg/mL (IQR: 3.33–22.43) vs. 11.58 pg/mL (IQR: 4.23–39.8), p = 0.456]. Similarly, baseline C-reactive protein (CRP) did not differ significantly [12.82 mg/L (IQR: 5.37–48.12) vs. 15.75 mg/L (IQR: 3.70–40.28), p = 0.734]. Preoperative carcinoembryonic antigen (CEA) levels were also comparable [4.77 ng/mL (IQR: 2.01–8.88) vs. 3.16 ng/mL (IQR: 1.78–8.87), p = 0.357]. The proportion of patients with elevated biomarkers at baseline was similar across groups (all p > 0.05), indicating balance in initial inflammatory and tumor burden status.

### 3.5 Tumor characteristics

Most tumors were moderately differentiated in both groups (86.4% vs. 88.2%, p = 0.712). Tumor location was evenly distributed: right colon (50.0% vs. 47.1%), left colon (31.8% vs. 35.3%), transverse colon (13.6% vs. 11.8%), and rectosigmoid (4.5% vs. 5.9%) (p = 0.956). Lymphovascular invasion was present in 45.5% of the Del-Immune V group versus 41.2% of the Placebo group (p = 0.789). Infiltration up to the serosa was the most frequent finding in both groups (63.6% vs. 64.7%, p = 0.834).

#### 3.5.1 TNM staging

The distribution of tumor stage was Stage I (4.5% vs. 0%), Stage II (18.2% vs. 23.5%), Stage III (54.5% vs. 64.7%), and Stage IV (22.7% vs. 0%). A trend toward a higher proportion of Stage IV disease was observed in the Del-Immune V group (p = 0.056), representing an important limitation since these patients had unresectable metastatic disease and were referred directly to oncology. When Stage III and IV were combined, the difference was not statistically significant (77.3% vs. 64.7%, p = 0.389).

#### 3.5.2 Surgical data and treatment

All patients were managed under the Enhanced Recovery After Surgery (ERAS) protocol (100% in both groups). Length of hospital stay tended to be longer in the Del-Immune V group [5.0 days (IQR: 3.0–7.0) vs. 3.0 days (IQR: 3.0–5.0), p = 0.070], likely explained by the higher proportion of advanced-stage cases. The reintervention rate was 18.2% in the intervention group versus 0% in the Placebo group (p = 0.056), mainly associated with Stage IV patients requiring additional management. Thirty-day mortality was low in both groups (4.5% vs. 0%, p = 1.000).

Adjuvant chemotherapy was administered to 68.2% of patients in the Del-Immune V group versus 76.5% in the placebo group (p = 0.736). The distribution of chemotherapy regimens (FOLFOX and XELOX) was balanced between groups (all p > 0.05).

### 3.6 Biomarkers and body mass index – pre- and postoperative levels

Biomarkers and BMI were evaluated at two time points: baseline (Week 0, preoperative) and follow-up (Week 8, approximately one month after surgery). Intra-group and inter-group comparisons are summarized in **Table 2**.

**Table 2:** Pre- and postoperative levels of inflammatory and oncological biomarkers (intra-group and inter-group comparisons).

Variable	Time period	Del-immune V (n=22)	Placebo (n=17)	P-value^a^ (inter)	P-value^b^ (intra)
BMI (kg/m²)	Baseline	24.6 (22.6-27.7)	25.8 (20.8-28.7)	0.867	0.234
	EOS	23.7 (22.4-26.5)	23.9 (19.8-26.2)	0.923	
	Abosolute Δ *	-0.5 (-1.8-0.3)	-0.4 (-1.5-0.6)	0.789	0.456
IL-6 (pg/mL)	Baseline	8.71 (3.33-22.43)	11.58 (4.23-39.8)	0.456	0.012
	EOS	4.23 (2.34-8.59)	6.23 (3.22-12.35)	0.189	
	Abosolute Δ *	-3.52 (-8.24-0.45)	-2.15 (-6.89-1.23)	0.634	0.570
CRP (mg/L)	Baseline	12.82 (5.37-48.12)	15.75 (3.70-40.28)	0.734	<0.001
	EOS	3.26 (2.06-6.28)	3.46 (2.27-8.64)	0.657	
	Abosolute Δ *	-8.54 (-25.3-(-2.1))	-5.89 (-18.7-(-1.5))	0.423	0.001
CEA (ng/mL)	Baseline	4.77 (2.01-8.88)	3.16 (1.78-8.87)	0.357	0.076
	EOS	2.95 (1.65-5.42)	2.49 (1.29-3.42)	0.312	
	Abosolute Δ *	-1.23 (-4.56-0.34)	-0.89 (-3.21-0.67)	0.567	0.155

*Data are expressed as median (interquartile range, IQR). EOS, End of Study. Δ = (Final – Baseline). a Intra-group p-value = comparison within the group (paired Wilcoxon test). b Inter-group p-value = comparison between groups (Mann–Whitney U test). BMI, body mass index; IL-6, interleukin-6; CRP, C-reactive protein; CEA, carcinoembryonic antigen; SD, standard deviation; IQR, interquartile range.

#### 3.6.1 Body mass index

Baseline BMI was comparable between the Del-Immune V and Placebo group [24.6 kg/m² (IQR: 22.6–27.7) vs. 25.8 kg/m² (IQR: 20.8–28.7), p = 0.867]. At Week 8, no significant differences were observed [23.7 kg/m² (IQR: 22.4–26.5) vs. 23.9 kg/m² (IQR: 19.8–26.2), p = 0.923].

The absolute change in BMI (Δ = Final – Baseline) was similar between groups [−0.5 kg/m² (IQR: −1.8–0.3) vs. −0.4 kg/m² (IQR: −1.5–0.6), p = 0.789]. Intra-group comparisons showed no significant changes from baseline to Week 8 (Del-Immune V: p = 0.234; Control: p > 0.05).

#### 3.6.2 Interleukin-6 (IL-6)

At Week 8, the Del-Immune V group showed lower IL-6 levels [4.23 pg/mL (IQR: 2.34–8.59)] compared to the Placebo group [6.23 pg/mL (IQR: 3.22–12.35)], though the difference was not statistically significant (p = 0.189).

Intra-group analysis revealed a significant reduction in IL-6 from baseline to Week 8 in the Del-Immune V group (p = 0.012), whereas no significant change was observed in the Placebo group (p = 0.570). The absolute change (Δ) was greater in the Del-Immune V group [−3.52 pg/mL (IQR: −8.24–0.45)] compared to Control [−2.15 pg/mL (IQR: −6.89–1.23)], without significant inter-group differences (p = 0.634). These results suggest that Del-Immune V may be associated with a significant reduction in IL-6, reflecting modulation of systemic inflammatory response.

#### 3.6.3 C-reactive protein

At Week 8, both groups showed marked reductions in CRP, with no significant differences between them [3.26 mg/L (IQR: 2.06–6.28) vs. 3.46 mg/L (IQR: 2.27–8.64), p = 0.657].

Intra-group analysis demonstrated highly significant reductions in CRP from baseline to Week 8 (Del-Immune V: p < 0.001; Control: p = 0.001). The absolute change was greater in the Del-Immune V group [−8.54 mg/L (IQR: −25.3–−2.1)] compared to Control [−5.89 mg/L (IQR: −18.7–−1.5)], though differences were not statistically significant (p = 0.423). These findings indicate that surgical resection itself induces a significant reduction in CRP, reflecting resolution of tumor-associated inflammation. The trend toward greater reduction in the Del-Immune V group may suggest an additive effect of the metabiotic.

#### 3.6.4 Carcinoembryonic antigen

At Week 8, both groups showed reductions in CEA levels, with no significant differences [2.95 ng/mL (IQR: 1.65–5.42) vs. 2.49 ng/mL (IQR: 1.29–3.42), p = 0.312].

Intra-group analysis showed a trend toward reduction in the Del-Immune V group (p = 0.076), while no significant change was observed in the Placebo group (p = 0.155). The absolute change was similar between groups [−1.23 ng/mL (IQR: −4.56–0.34) vs. −0.89 ng/mL (IQR: −3.21–0.67), p = 0.567]. These results suggest that tumor resection is associated with postoperative reductions in CEA, as expected. The trend toward greater reduction in the Del-Immune V group may indicate a beneficial effect on residual tumor burden or cancer-associated inflammation.

#### 3.6.5 Magnitude of percent change in biomarkers

The magnitude of change in inflammatory and oncological biomarkers was evaluated by calculating the percent change (Δ%) between baseline values (Week 0) and final values (Week 8). In addition, the proportion of patients achieving normalization of each biomarker (values within the reference range at the end of follow-up) was analyzed. Results are summarized in **Table 3**.

**Table 3:** Magnitude of percent change in biomarkers.

Biomarker	Del-immune V (n=22)	Placebo (n=17)	P-valor
Δ IL-6 (%)	-45.2% (-68.5-(-12.3))	-28.7% (-55.4-8.9)	0.234*^1^*
Δ PCR (%)	-62.8% (-85.2-(-35.6))	-48.3% (-72.1-(-18.9))	0.189*^1^*
Δ CEA (%)	-32.5% (-58.9-12.4)	-25.6% (-48.7-18.3)	0.445*^1^*
Patients with IL-6 normalization, n (%)	14 (63.6%)	8 (47.1%)	0.298*^2^*
Patients with CRP normalization, n (%)	18 (81.8%)	12 (70.6%)	0.489*^2^*
Patients with CEA normalization, n (%)	15 (68.2%)	11 (64.7%)	0.812*^2^*

Data are expressed as median (interquartile range, IQR). Normalization criteria: IL-6 <5 pg/mL, CRP <5 mg/L, CEA <4.7 ng/mL. ¹ Mann–Whitney U test; ² Fisher’s exact test.

#### 3.6.6 Percent change in interleukin-6

Percent change in IL-6 levels showed a greater reduction in the Del-Immune V group [−45.2% (IQR: −68.5 to −12.3)] compared to the Placebo group [−28.7% (IQR: −55.4 to 8.9)], although the difference did not reach statistical significance (p = 0.234). Notably, the interquartile range in the Placebo group included positive values (up to 8.9%), indicating that some patients experienced an increase in IL-6 rather than a reduction.

#### 3.6.7 Percent change in C-reactive protein

Percent change in CRP demonstrated a more pronounced reduction in the Del-Immune V group [−62.8% (IQR: −85.2 to −35.6)] compared to the Placebo group [−48.3% (IQR: −72.1 to −18.9)], with differences not reaching statistical significance (p = 0.189). The magnitude of CRP reduction exceeded 60% in the intervention group, reflecting faster normalization of this acute-phase inflammatory marker.

#### 3.6.8 Percent change in carcinoembryonic antigen

Percent change in CEA was similar between groups [−32.5% (IQR: −58.9 to 12.4) vs. −25.6% (IQR: −48.7 to 18.3), p = 0.445]. Both groups showed moderate reductions consistent with surgical tumor resection. Interquartile ranges included positive values in both groups, indicating that a subset of patients experienced increases in CEA, potentially reflecting residual or progressive disease.

#### 3.6.9 Biomarker normalization rates

IL-6: Normalization (<5 pg/mL at Week 8) occurred in 14 of 22 patients (63.6%) in the Del-Immune V group versus 8 of 17 patients (47.1%) in the Placebo group (p = 0.298).

CRP: Normalization (<5 mg/L at Week 8) was achieved in 18 of 22 patients (81.8%) in the Del-Immune V group versus 12 of 17 patients (70.6%) in the Placebo group (p = 0.489).

CEA: Normalization (<4.7 ng/mL at Week 8) was observed in 15 of 22 patients (68.2%) in the Del-Immune V group versus 11 of 17 patients (64.7%) in the Placebo group (p = 0.812).

### 3.7 Adverse effects

Fifteen patients (68.2%) in the Del-Immune V group reported at least one adverse effect, compared with 11 patients (64.7%) in the placebo group, with no statistically significant differences (p = 0.812). Total adverse events were 20 vs. 14 (p = 0.456).Mean adverse events per patient were 0.91 vs. 0.82 (p = 0.734), indicating similar burden across groups. (**Table 4**).

**Table 4:** Adverse effects by treatment group.

Advance effect	Severity	Del-immune V (n=22)	Placebo (n=17)	P-value
Gastrointestinal pain/discomfort	Mild	3 (13.6%)	1 (5.9%)	0.356
	Moderate	1 (4.5%)	0 (0%)	
	Total	4 (18.2%)	1 (5.9%)	
Gas	Mild	3 (13.6%)	3 (17.6%)	0.736
	Moderate	1 (4.5%)	1 (5.9%)	
	Total	4 (18.2%)	4 (23.5%)	
Anxiety/depression	Mild	1 (4.5%)	0 (0%)	1.000
	Severe	0 (0%)	1 (5.9%)	
	Total	1 (4.5%)	1 (5.9%)	
Joint pain	Mild	0 (0%)	2 (11.8%)	0.206
	Total	0 (0%)	2 (11.8%)	
Diarrhea	Mild	2 (9.1%)	0 (0%)	0.634
	Moderate	0 (0%)	1 (5.9%)	
	Severe	1 (4.5%)	0 (0%)	
	Total	3 (13.6%)	1 (5.9%)	
Constipation	Mild	1 (4.5%)	1 (5.9%)	0.577
	Moderate	0 (0%)	1 (5.9%)	
	Total	1 (4.5%)	2 (11.8%)	
Headache	Mild	2 (9.1%)	0 (0%)	0.234
	Moderate	1 (4.5%)	0 (0%)	
	Total	3 (13.6%)	0 (0%)	
Hemorrhoids	Mild	0 (0%)	1 (5.9%)	0.206
	Moderate	0 (0%)	1 (5.9%)	
	Total	0 (0%)	2 (11.8%)	
Migraine	Mild	1 (4.5%)	0 (0%)	1.000
	Total	1 (4.5%)	0 (0%)	
Insomnia	Mild	2 (9.1%)	0 (0%)	0.634
	Moderate	1 (4.5%)	0 (0%)	
	Severe	0 (0%)	1 (5.9%)	
	Total	3 (13.6%)	1 (5.9%)	

p-values for comparisons of adverse effect proportions between groups were calculated using Fisher’s exact test, appropriate for small samples and cells with expected frequencies <5. The significance level was set at α = 0.05. All adverse effects were analyzed as binary variables (presence/absence), regardless of severity, for the total incidence analysis. No adverse effects were directly related to capsule intake in either group (0%), indicating good overall tolerability of the intervention.

Most adverse effects were mild in both groups, with Del−Immune V reporting 15 mild (75.0%), 4 moderate (20.0%), and 1 severe (5.0%) events, while the placebo group reported 10 mild (71.4%), 3 moderate (21.4%), and 1 severe (7.1%) events, with no significant differences in severity distribution (p = 1.000). Regarding specific adverse effects, gastrointestinal discomfort was more frequent in the Del−Immune V group (18.2% vs. 5.9%, p = 0.356), gas was similar between groups (18.2% vs. 23.5%, p = 0.736), diarrhea was slightly higher in Del−Immune V (13.6% vs. 5.9%, p = 0.634), and constipation was more frequent in placebo (11.8% vs. 4.5%, p = 0.577). Neurological events included headache, observed exclusively in Del−Immune V (13.6% vs. 0%, p = 0.234), migraine (4.5% vs. 0%, p = 1.000), and insomnia, more frequent in Del−Immune V (13.6% vs. 5.9%, p = 0.634). Musculoskeletal events included joint pain, reported only in placebo (11.8% vs. 0%, p = 0.206). Psychiatric symptoms such as anxiety/depression were similar between groups (4.5% vs. 5.9%, p = 1.000), while hemorrhoids were reported exclusively in placebo (11.8% vs. 0%, p = 0.206). Importantly, no adverse effects were directly related to capsule intake in either group (0%), indicating good overall tolerability of the intervention. (**Table 4**).

### 3.8 Beneficial effects

Overall, eighteen patients (81.8%) in the Del−Immune V group reported at least one beneficial effect compared to fifteen patients (88.2%) in the placebo group (p = 0.701), with a total of 49 vs. 46 beneficial events (p = 0.678) and a mean of 2.23 vs. 2.71 events per patient (p = 0.456) (**Table 5**). Regarding specific beneficial effects, improvements in intestinal function included similar reductions in intestinal gas (22.7% vs. 29.4%, p = 0.736), slightly lower improvements in bowel habits in Del−Immune V (22.7% vs. 35.3%, p = 0.489), and fewer reports of soft, molded stools (22.7% vs. 41.2%, p = 0.289). Digestive function outcomes were comparable between groups, with similar increases in appetite (31.8% vs. 35.3%, p = 0.812), improved digestion (27.3% vs. 35.3%, p = 0.736), and reduced gastric acidity (18.2% vs. 17.6%, p = 1.000). Quality−of−life measures showed similar increases in physical energy (27.3% vs. 29.4%, p = 1.000) and faster recovery from surgery (31.8% vs. 35.3%, p = 0.812), while reduced anxiety was more frequent in the Del−Immune V group (18.2% vs. 5.9%, p = 0.356), with an absolute difference of 12.3% in favor of the intervention. Other beneficial effects included rectal bleeding cessation, which was reported exclusively in the placebo group (5.9% vs. 0%, p = 0.487) (**Table 5**).

**Table 5:** Beneficial effects by treatment group.

Beneficial effects	Del-Immune V (n=22)	Placebo (n=17)	p-value
Reduction of intestinal gas	5 (22.7%)	5 (29.4%)	0.736
Improved bowel habits	5 (22.7%)	6 (35.3%)	0.489
Soft, molded stools	5 (22.7%)	7 (41.2%)	0.289
Increased appetite	7 (31.8%)	6 (35.3%)	0.812
Improved digestion	6 (27.3%)	6 (35.3%)	0.736
Reduced gastric acidity	4 (18.2%)	3 (17.6%)	1.000
Increased physical energy	6 (27.3%)	5 (29.4%)	1.000
Rapid postoperative recovery	7 (31.8%)	6 (35.3%)	0.812
Reduced anxiety	4 (18.2%)	1 (5.9%)	0.356
Rectal bleeding cessation	0 (0%)	1 (5.9%)	0.487
Total beneficial effects	49 eventos	46 eventos	0.678
Patients with ≥1 beneficial effect	18 (81.8%)	15 (88.2%)	0.701

p-values for comparisons of beneficial effect proportions between groups were calculated using Fisher’s exact test, appropriate for small samples and cells with expected frequencies <5. The significance level was set at α = 0.05.

### 3.9 Health-related quality of life analysis (EORTC QLQ-C30)

#### 3.9.1 Sample characteristics

Health-related quality of life was assessed using the EORTC QLQ-C30 questionnaire (version 3) at two points: baseline (preoperative/start of treatment) and endpoint (approximately 8 weeks postoperative). A total of 39 patients completed both assessments: 22 in the Del-Immune V group (Batch 426) and 17 in the placebo group (Batch 425), representing a 100% retention rate in both groups.

#### 3.9.2 Functional scales

Significant intra−group improvements were observed in the Del−Immune V group across several domains, including physical function (+5.8 points, p = 0.034), role function (+6.8 points, p = 0.028), emotional function (+7.3 points, p = 0.018), social function (+6.8 points, p = 0.042), and global quality of life (+8.4 points, p = 0.012). In contrast, the placebo group did not show statistically significant changes in these areas (physical function +2.5 points, p = 0.189; role function +3.1 points, p = 0.267; emotional function +2.1 points, p = 0.456; social function +2.3 points, p = 0.312; global quality of life +3.0 points, p = 0.178). No significant changes were observed in cognitive function in either group. (**Table 6**).

**Table 6:** Functional scale scores – baseline and endpoint.

Functional scale	Time point	Del-immune V (n=22)	Placebo (n=17)	P-valor (inter)	P-valor (intra)
Physical Function	baseline	72.5 (58.3-83.3)	70.8 (62.5-81.3)	0.823	0.034*
	EOS	78.3 (66.7-87.5)	73.3 (64.6-82.3)	0.456	0.189
	Δ (EOS- baseline)	+5.8 (-2.5-15.0)	+2.5 (-5.0-10.0)	0.234	
Role Function	baseline	68.2 (50.0-83.3)	66.7 (54.2-79.2)	0.912	0.028*
	EOS	75.0 (62.5-87.5)	69.8 (58.3-81.3)	0.378	0.267
	Δ (EOS- baseline)	+6.8 (-1.7-16.7)	+3.1 (-4.2-11.5)	0.312	
Emotional Function	baseline	64.6 (52.1-77.1)	63.5 (54.2-75.0)	0.867	0.018*
	EOS	71.9 (60.4-83.3)	65.6 (56.3-76.0)	0.289	0.456
	Δ (EOS- baseline)	+7.3 (-0.8-15.6)	+2.1 (-5.2-9.4)	0.178	
Cognitive Function	baseline	70.8 (58.3-83.3)	69.6 (60.4-80.2)	0.934	0.156
	EOS	74.2 (64.6-85.4)	71.5 (62.5-81.3)	0.512	0.389
	Δ (EOS- baseline)	+3.4 (-3.3-10.4)	+1.9 (-4.2-8.3)	0.445	
Función SocialSocial Function	baseline	66.7 (54.2-79.2)	65.4 (56.3-77.1)	0.889	0.042*
	EOS	73.5 (62.5-84.4)	67.7 (58.3-78.1)	0.334	0.312
	Δ (EOS- baseline)	+6.8 (-1.0-14.6)	+2.3 (-4.6-9.2)	0.223	
Global Quality of Life	baseline	58.3 (47.9-68.8)	57.4 (50.0-66.7)	0.945	0.012*
	EOS	66.7 (56.3-77.1)	60.4 (52.1-69.8)	0.267	0.178
	Δ (EOS- baseline)	+8.4 (0.0-16.7)	+3.0 (-3.1-9.1)	0.156	

Data are expressed as median (interquartile range, IQR). Inter-group p-values were calculated using the Mann–Whitney U test. All other p-values refer to within-group variations (endpoint vs. baseline), assessed with the Wilcoxon signed-rank test.

#### 3.9.3 Symptom scales

In the Del−Immune V group, significant reductions were observed in several symptom domains, including fatigue (−6.7 points, p = 0.024), pain (−7.2 points, p = 0.032), and insomnia (−7.3 points, p = 0.018), whereas the placebo group did not show statistically significant changes in these areas (fatigue −2.4 points, p = 0.156; pain −2.7 points, p = 0.178; insomnia −1.9 points, p = 0.456). Although other symptoms did not reach statistical significance, the trends consistently favored Del−Immune V, suggesting a potential therapeutic benefit across multiple dimensions of patient well−being. (**Table 7**).

**Table 7:** Symptom scale scores – baseline and endpoint.

Symptom	Time point	Del-immune V (n=22)	Placebo (n=17)	P-value (inter)	P-value (intra)
Fatigue	baseline	42.5 (33.3-54.2)	44.1 (35.4-53.1)	0.812	0.024*
	EOS	35.8 (27.1-45.8)	41.7 (33.3-51.0)	0.234	0.156
	Δ (EOS- baseline)	-6.7 (-14.6-1.3)	-2.4 (-9.4-4.6)	0.267	
Nausea/Vomiting	baseline	18.8 (10.4-29.2)	20.6 (12.5-30.2)	0.734	0.089
	EOS	14.6 (8.3-22.9)	18.6 (11.5-27.1)	0.312	0.234
	Δ (EOS- baseline)	-4.2 (-10.4-2.1)	-2.0 (-7.3-3.1)	0.389	
Pain	baseline	38.5 (29.2-50.0)	40.2 (31.3-49.0)	0.867	0.032*
	EOS	31.3 (22.9-41.7)	37.5 (29.2-46.9)	0.289	0.178
	Δ (EOS- baseline)	-7.2 (-14.6-0.4)	-2.7 (-8.3-3.1)	0.234	
Dyspnea	baseline	25.0 (16.7-35.4)	26.5 (18.8-34.4)	0.923	0.145
	EOS	21.9 (14.6-30.2)	25.0 (17.7-33.3)	0.456	0.267
	Δ (EOS- baseline)	-3.1 (-9.4-3.1)	-1.5 (-6.3-4.2)	0.378	
Insomnia	baseline	35.4 (25.0-47.9)	36.3 (27.1-45.8)	0.889	0.018*
	EOS	28.1 (19.8-38.5)	34.4 (26.0-43.8)	0.312	0.456
	Δ (EOS- baseline)	-7.3 (-14.6-0.0)	-1.9 (-7.3-3.6)	0.189	
Loss of Appetite	baseline	28.1 (18.8-39.6)	29.4 (20.8-38.5)	0.845	0.067
	EOS	22.9 (15.6-32.3)	27.5 (19.8-36.5)	0.378	0.234
	Δ (EOS- baseline)	-5.2 (-11.5-1.0)	-1.9 (-6.3-2.6)	0.267	
Constipation	baseline	31.3 (22.9-42.7)	32.4 (24.0-41.7)	0.912	0.112
	EOS	26.0 (18.8-35.4)	30.4 (22.9-39.6)	0.423	0.289
	Δ (EOS- baseline)	-5.3 (-12.5-1.9)	-2.0 (-7.3-3.1)	0.312	
Diarrhea	baseline	22.9 (15.6-32.3)	24.5 (17.7-31.3)	0.867	0.134
	EOS	19.8 (13.5-28.1)	23.5 (16.7-30.2)	0.389	0.312
	Δ (EOS- baseline)	-3.1 (-8.3-2.1)	-1.0 (-5.2-3.1)	0.345	
Financial Difficulties	baseline	38.5 (29.2-50.0)	39.2 (30.2-48.0)	0.934	0.098
	EOS	34.4 (26.0-44.8)	37.5 (29.2-46.9)	0.467	0.267
	Δ (EOS- baseline)	-4.1 (-10.4-2.1)	-1.7 (-6.3-2.6)	0.334	

Data are expressed as median (interquartile range, IQR). Inter-group p-values were calculated using the Mann–Whitney U test. All other p-values refer to within-group variations (endpoint vs. baseline), assessed with the Wilcoxon signed-rank test.

#### 3.9.4 Colorectal cancer–specific symptoms

In the Del−Immune V group, significant reductions were observed in abdominal pain (−7.3 points, p = 0.028) and blood in stools (−7.3 points, p = 0.034), whereas the placebo group did not show statistically significant changes in these outcomes (abdominal pain −3.0 points, p = 0.178; blood in stools −2.0 points, p = 0.312). These findings suggest that Del−Immune V may provide meaningful symptom relief compared to placebo. (**Table 8**).

**Table 8:** Colorectal cancer–specific symptom scores – baseline and endpoint.

Specific symptom	Time point	Del-immune V (n=22)	Placebo (n=17)	P-value (inter)	P-value (intra)
Abdominal Pain	baseline	35.4 (26.0-46.9)	36.3 (28.1-44.8)	0.878	0.028*
	EOS	28.1 (20.8-37.5)	33.3 (26.0-42.7)	0.312	0.178
	Δ (EOSl- baseline)	-7.3 (-14.6-0.0)	-3.0 (-8.3-2.1)	0.245	
Anal/Rectal Pain	baseline	28.1 (19.8-38.5)	29.4 (21.9-37.5)	0.845	0.045*
	EOS	22.9 (16.7-31.3)	27.5 (20.8-35.4)	0.378	0.234
	Δ (EOSl- baseline)	-5.2 (-11.5-1.0)	-1.9 (-6.3-2.6)	0.289	
Blood in Stools	baseline	31.3 (22.9-41.7)	32.4 (24.0-40.6)	0.912	0.034*
	EOS	24.0 (17.7-32.3)	30.4 (23.0-38.5)	0.267	0.312
	Δ (EOSl- baseline)	-7.3 (-13.5-(-1.0))	-2.0 (-7.3-2.6)	0.198	
Mucus in Stools	baseline	26.0 (18.8-35.4)	27.5 (19.8-34.4)	0.867	0.078
	EOS	21.9 (15.6-30.2)	25.5 (18.8-33.3)	0.423	0.267
	Δ (EOSl- baseline)	-4.1 (-9.4-1.0)	-2.0 (-6.3-2.1)	0.334	
Dry Mouth	baseline	32.3 (24.0-42.7)	33.3 (25.0-41.7)	0.923	0.089
	EOS	27.1 (20.8-35.4)	31.4 (24.0-39.6)	0.389	0.234
	Δ (EOSl- baseline)	-5.2 (-10.4-0.0)	-1.9 (-6.3-2.6)	0.278	
Taste Problems	baseline	29.2 (21.9-38.5)	30.4 (22.9-37.5)	0.889	0.067
	EOS	24.0 (17.7-32.3)	28.5 (21.9-36.5)	0.356	0.198
	Δ (EOSl- baseline)	-5.2 (-10.4-0.0)	-1.9 (-5.2-1.6)	0.256	

Data are expressed as median (interquartile range, IQR). Inter-group p-values were calculated using the Mann–Whitney U test. All other p-values refer to within-group variations (endpoint vs. baseline), assessed with the Wilcoxon signed-rank test.

### 3.10 Microbiome analysis

Compositional changes in the gut microbiome were evaluated using 16S rRNA gene sequencing of stool samples collected at baseline and end-of-study (EOS) from participants randomized to Del-Immune V or placebo. Sequence data were processed using a standardized bioinformatics pipeline to generate amplicon sequence variants and corresponding taxonomic assignments, after which diversity metrics were calculated at the rarefied feature table level. Microbial community structure was assessed at multiple levels, including within-sample (alpha) diversity, between-sample (beta) diversity, and taxonomic composition, to determine whether Del-Immune V supplementation produced measurable shifts in gut microbiota organization relative to placebo.

Alpha diversity indices (Chao richness estimator, Shannon index, and Simpson index) were used to capture complementary aspects of microbial richness, overall diversity, and community evenness over the intervention period. These metrics allowed us to distinguish between simple loss of taxes and more nuanced restructuring of the community, including changes in the relative contribution of low-abundance and dominant taxa.

#### 3.10.1 Alpha diversity

**Figure 2:**
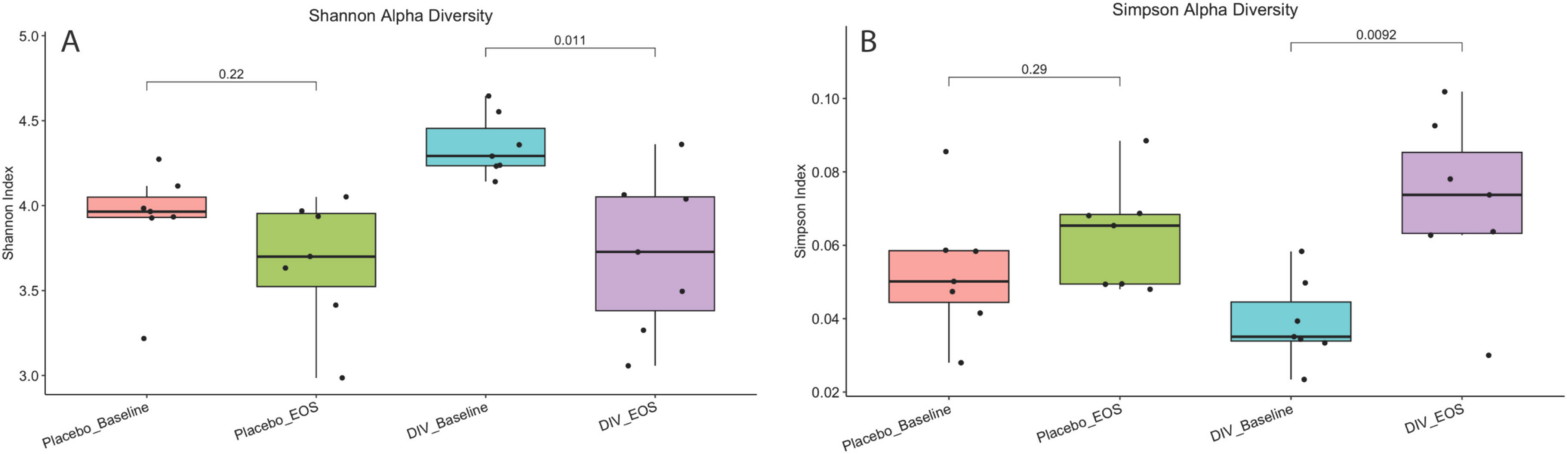
Effects of del-immune V on gut microbiome alpha diversity. Box-and-whisker plots illustrate the distribution of the **(A)** Shannon and **(B)** Simpson diversity indices for fecal samples collected at baseline and end-of-study (EOS). Data are derived from a microbiome subset of n = 17 patients (n = 7 for Del-Immune V; n = 10 for Placebo). In the boxplots, the central horizontal line represents the median, boxes represent the interquartile range (IQR), whiskers extend to the most extreme values within 1.5 × IQR, and overlaid jittered dots represent individual participant values. Intra-group comparisons (Baseline vs. EOS) were performed using the Wilcoxon signed-rank test. Inter-group comparisons (Del-Immune V vs. Placebo at EOS) were assessed using the Mann-Whitney U test. Statistical significance was defined as *p* < 0.05. *p*-values are displayed above the corresponding comparison brackets.

Alpha diversity analyses demonstrated modest but consistent restructuring of the gut microbiome over the intervention period in both the Del-Immune V and placebo cohorts (**Table 9**, **Figure 2**). Estimated richness (Chao index) decreased over time in each group, whereas Simpson diversity increased, especially in the Del-Immune V cohort, indicating a shift toward greater community evenness and reduced dominance by a small number of taxa. Shannon diversity showed a more modest decline, suggesting a reduction in low-abundance tax rather than a broad collapse of overall diversity. Collectively, these findings are more consistent with consolidation of the microbial community structure than with simple diversity loss.

**Table 9:** Mean alpha diversity indices (± standard deviation) for each cohort and timepoint.

Cohort	CHAO (mean ± SD)	Shannon (mean ± SD)	Simpson (mean ± SD)
DIV_Baseline	837.25 ± 137.4	4.05 ± 0.39	0.036 ± 0.012
DIV_EOS	694.16 ± 121.5	3.63 ± 0.33	0.075 ± 0.023
Placebo_Baseline	668.94 ± 109.8	3.67 ± 0.35	0.050 ± 0.018
Placebo_EOS	648.47 ± 92.6	3.59 ± 0.31	0.066 ± 0.015

Alpha diversity analyses showed modest but consistent restructuring: richness decreased while Simpson diversity increased, particularly in the Del-Immune V^®^ group, indicating greater community evenness and reduced dominance. Shannon diversity declined slightly, consistent with consolidation of microbial structure rather than overall diversity loss.

#### 3.10.2 Taxonomic distribution

**Figure 3:**
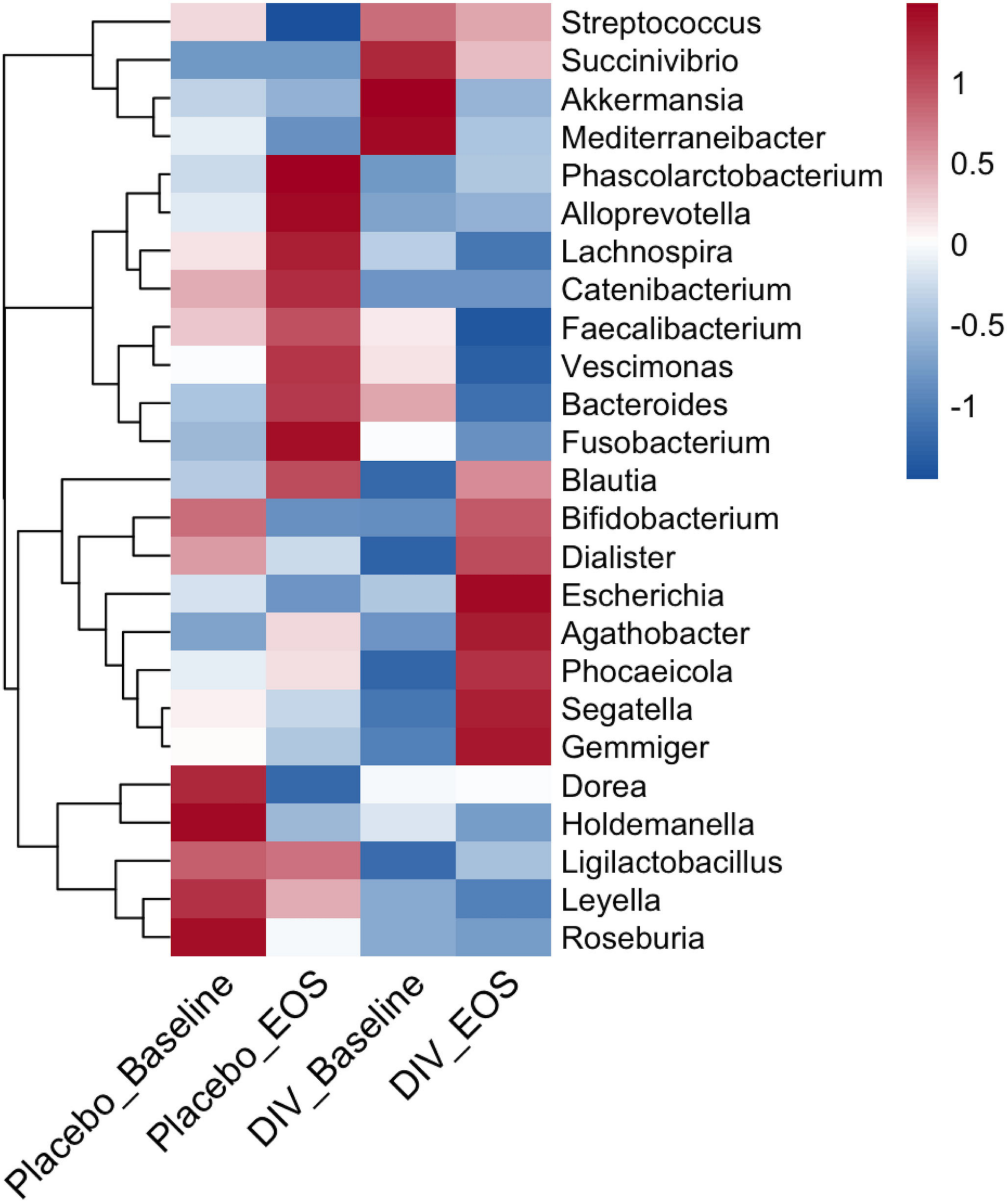
Heatmap illustrating genus-level gut microbiome restructuring. The heatmap displays row-scaled (z-score) relative abundances of the 25 most relevant bacterial genera across four conditions: Del-Immune V baseline (DIV_Baseline), Del-Immune V EOS (DIV_EOS), Placebo baseline (Placebo_Baseline), and Placebo EOS (Placebo_EOS). Data represent the mean relative abundances for the microbiome subset of n = 17 patients (n = 7 for Del-Immune V; n = 10 for Placebo). Color intensity indicates standardized abundance, with red representing higher and blue representing lower relative abundance compared to the overall mean. Genera were selected based on significant differential abundance shifts identified via LEfSe (Linear Discriminant Analysis Effect Size) and Kruskal-Wallis tests, with a significance threshold of *p* < 0.05 and an LDA score > 2.0. Hierarchical clustering (left dendrogram) highlights taxa co-varying across treatment and time.

Taxonomic profiling highlighted shifts in several dominant and subdominant genera across study arms and timepoints (**Figure 3**; **Table 10**). In the Del-Immune V cohort, multiple carbohydrate-fermenting and butyrate-associated taxa changed in relative abundance, whereas the placebo group showed more modest or divergent trends across the same genera.

**Table 10:** Key genera associated with microbiome restructuring in Del-Immune V and placebo cohorts.

Genus	DIV baseline	DIV EOS	Placebo baseline	Placebo EOS	Direction in DIV	Functional interpretation
*Segatella*	10.12	21.22	15.61	13.84	↑	Prevotellaceae fermenter associated with carbohydrate metabolism
*Faecalibacterium*	6.10	2.27	6.50	8.22	↓	Butyrate producer; reduced in DIV cohort
*Phocaeicola*	3.26	6.75	4.85	5.30	↑	Bacteroidaceae carbohydrate fermenter
*Blautia*	3.14	4.71	3.86	5.04	↑	Lachnospiraceae commensal linked to SCFA production
*Bacteroides*	4.93	2.23	3.41	6.05	↓	Major polysaccharide degrader
*Leyella*	1.91	1.55	3.73	2.99	↓	Minor fermentative genus
*Roseburia*	1.18	0.95	4.82	2.28	↓	Butyrate-producing Lachnospiraceae
*Phascolarctobacterium*	1.50	1.80	1.93	3.29	↑	Succinate-utilizing fermenter
*Akkermansia*	5.33	0.58	1.14	0.53	↓	Mucin-degrading specialist
*Streptococcus*	2.37	2.16	2.00	0.91	≈	Oral-associated facultative anaerobe
*Bifidobacterium*	0.75	2.70	2.58	0.76	↑	Beneficial saccharolytic commensal
*Escherichia*	1.11	3.55	1.37	0.54	↑	Facultative anaerobe responding to niche shifts
*Lachnospira*	1.35	0.83	1.73	2.56	↓	Fiber fermenter
*Succinivibrio*	3.88	2.23	0.03	0.03	↓	Succinate-producing fermenter
*Ligilactobacillus*	0.10	0.88	2.31	2.18	↑	Lactic acid bacterium
*Mediterraneibacter*	2.16	1.11	1.29	0.89	↓	Firmicutes fermenter
*Agathobacter*	0.58	2.52	0.67	1.49	↑	Butyrate-associated Lachnospiraceae
*Dorea*	1.28	1.29	1.68	0.92	≈	Common gut commensal
*Alloprevotella*	1.01	1.05	1.20	1.72	≈	Prevotellaceae fermenter
*Vescimonas*	1.22	0.55	1.15	1.68	↓	Minor anaerobic genus
*Catenibacterium*	0.71	0.72	1.34	1.70	≈	Fermentative Firmicutes
*Gemmiger*	0.54	1.79	1.09	0.85	↑	SCFA-producing Ruminococcaceae
*Fusobacterium*	1.02	0.41	0.65	2.04	↓	CRC-associated pathogen
*Holdemanella*	0.84	0.36	2.15	0.55	↓	Firmicutes anaerobe
*Dialister*	0.49	1.33	1.16	0.86	↑	Anaerobic fermenter associated with dysbiosis dynamics

Genus-level analysis showed microbiome restructuring in the Del-Immune V group, with increases in *Bifidobacterium, Agathobacter, Gemmiger*, and *Phocaeicola*, alongside a decline in CRC-associated Fusobacterium, whereas placebo samples retained or expanded dysbiosis-associated genera.

At the genus level, Del-Immune V was associated with increased *Segatella, Phocaeicola, Blautia, Phascolarctobacterium, Bifidobacterium, Escherichia, Ligilactobacillus*, and *Agathobacter* from baseline to EOS, with little or no corresponding increase in the placebo arm for several of these taxa. In contrast, key butyrate producers and fermentative Firmicutes including *Faecalibacterium, Roseburia, Lachnospira, Mediterraneibacter, Leyella*, and *Succinivibrio* decreased in the Del-Immune V group, while often remaining stable or increasing under placebo.

*Akkermansia*, a mucin-degrading specialist frequently linked to mucosal metabolism, declined sharply in the Del-Immune V arm (from 5.33 to 0.58) but remained low and stable in placebo. Genera such as *Streptococcus* and *Dorea* showed minimal net change in Del-Immune V, whereas several genera displayed opposite directions of change between arms (for example, *Bacteroides* decreased in Del-Immune V but increased in placebo). Quantitative relative abundances and directional trends for the genera highlighted in **Figure 3** are detailed in **Table 10**, along with functional annotations for each taxon.

#### 3.10.3 Microbiome reconstruction

Genus-level analysis identified coordinated restructuring of the gut microbiome in the Del-Immune V cohort. Notable increases were observed in *Bifidobacterium*, *Agathobacter*, *Gemmiger*, and *Phocaeicola*, while CRC-associated taxa such as *Fusobacterium* declined relative to baseline. In contrast, placebo samples showed persistence or expansion of several dysbiosis-associated genera (**Table 10**).

#### 3.10.4 Functional guild analysis

Functional guild analysis revealed clear differences in microbial community organization between the placebo and Del-Immune V groups at the end of the study. **Table 11** summarizes the overall guild structure.

**Table 11:** Functional guild structure of key genera.

Functional guild	Genera	Ecological role	Direction in DIV
Carbohydrate Fermenters (Primary degraders)	*Segatella*, *Phocaeicola*, *Bacteroides*, *Alloprevotella*	Degradation of complex polysaccharides and dietary fibers	Mixed; enrichment of Segatella and Phocaeicola
SCFA-Producing Commensals	*Bifidobacterium*, *Agathobacter*, *Gemmiger*, *Blautia*, *Roseburia*, *Faecalibacterium*	Production of acetate and butyrate supporting epithelial health	Enrichment of Bifidobacterium, Agathobacter, Gemmiger
Secondary Fermenters/Cross-feeders	*Phascolarctobacterium*, *Dialister*, *Dorea*, *Mediterraneibacter*, *Catenibacterium*	Utilize fermentation intermediates such as lactate and succinate	Moderate shifts
Lactic Acid Producers	*Ligilactobacillus*, *Streptococcus*	Produce lactate used by secondary fermenters	Slight enrichment
Mucin Specialists	*Akkermansia*	Mucin degradation and host–microbe interface interactions	Decline observed
Opportunistic/Dysbiosis-Associated Taxa	*Escherichia*, *Fusobacterium*	Facultative anaerobes associated with inflammation or CRC	*Fusobacterium* decreased
Low-Abundance Anaerobes	*Vescimonas*, *Holdemanella*, *Leyella*	Minor fermentative roles within the gut ecosystem	Variable

Functional guild analysis at EOS revealed distinct community structures: placebo microbiomes were dominated by carbohydrate degraders and SCFA producers but retained CRC-associated Fusobacterium, whereas Del-Immune V^®^ promoted expansion of degraders and SCFA commensals with a marked decline in Fusobacterium.

Functional guild analysis revealed clear differences in microbial community organization between the placebo and Del-Immune V groups at the end of the study. In the placebo EOS microbiome (**Table 12**), the community was dominated by primary carbohydrate degraders, including *Segatella* (13.84%), *Bacteroides* (6.05%), and *Phocaeicola* (5.30%). A strong SCFA-producing guild was present, led by *Faecalibacterium* (8.22%), with additional contributions from *Blautia* (5.04%) and *Roseburia* (2.28%). Secondary fermenters such as *Phascolarctobacterium* and *Lachnospira* formed a moderate cross-feeding network supporting short-chain fatty acid production. However, the placebo microbiome also contained CRC-associated taxa, most notably *Fusobacterium* (2.04%), indicating persistence of dysbiosis-associated organisms despite otherwise stable fermentative guilds.

**Table 12:** Functional guild structure – placebo vs. del-immune V microbiome at end of study.

Genera	Placebo (%)	Del-immune V (%)	Delta (pp)	% change vs placebo
Primary carbohydrate degraders				
Segatella	13.84	21.22	+7.38	+53.3%
Phocaeicola	5.30	6.75	+1.45	+27.4%
Bacteroides	6.05	2.23	-3.82	-63.1%
Alloprevotella	1.72	1.05	-0.67	-39.0%
SCFA producers/SCFA-associated commensals				
Faecalibacterium	8.22	–	–	–
Blautia	5.04	4.71	-0.33	-6.5%
Roseburia	2.28	–	–	–
Agathobacter	1.49	2.52	+1.03	+69.1%
Gemmiger	0.85	1.79	+0.94	+110.6%
Bifidobacterium	–	2.70	–	–
Secondary fermenters				
Phascolarctobacterium	3.29	1.80	-1.49	-45.3%
Lachnospira	2.56	–	–	–
Dialister	0.86	1.33	+0.47	+54.7%
Dorea	0.92	1.29	+0.37	+40.2%
Lactic acid producers				
Streptococcus	0.91	2.16	+1.25	+137.4%
Ligilactobacillus	2.18	0.88	-1.30	-59.6%
Succinivibrio	–	2.23	–	–
Mucin specialists				
Akkermansia	0.53	0.58	+0.05	+9.4%
CRC-associated taxa				
Fusobacterium	2.04	0.41	-1.63	-79.9%
Minor anaerobes				
Leyella	2.99	1.55	-1.44	-48.2%
Vescimonas	1.68	0.55	-1.13	-67.3%
Holdemanella	0.55	0.36	-0.19	-34.5%

In contrast, the Del-Immune V EOS microbiome showed a notable restructuring of functional guilds. The primary carbohydrate degrader guild expanded, with *Segatella* increasing to 21.22% and *Phocaeicola* rising to 6.75%. The community also showed enrichment of SCFA-associated commensals, including *Bifidobacterium* (2.70%), *Agathobacter* (2.52%), and *Gemmiger* (1.79%), suggesting enhanced metabolic cross-feeding within the fermentation network. At the same time, the relative abundance of CRC-associated taxa decreased substantially, with *Fusobacterium* declining to 0.41% (**Table 12**).

#### 3.10.5 Dysbiotic index

**Figure 4:**
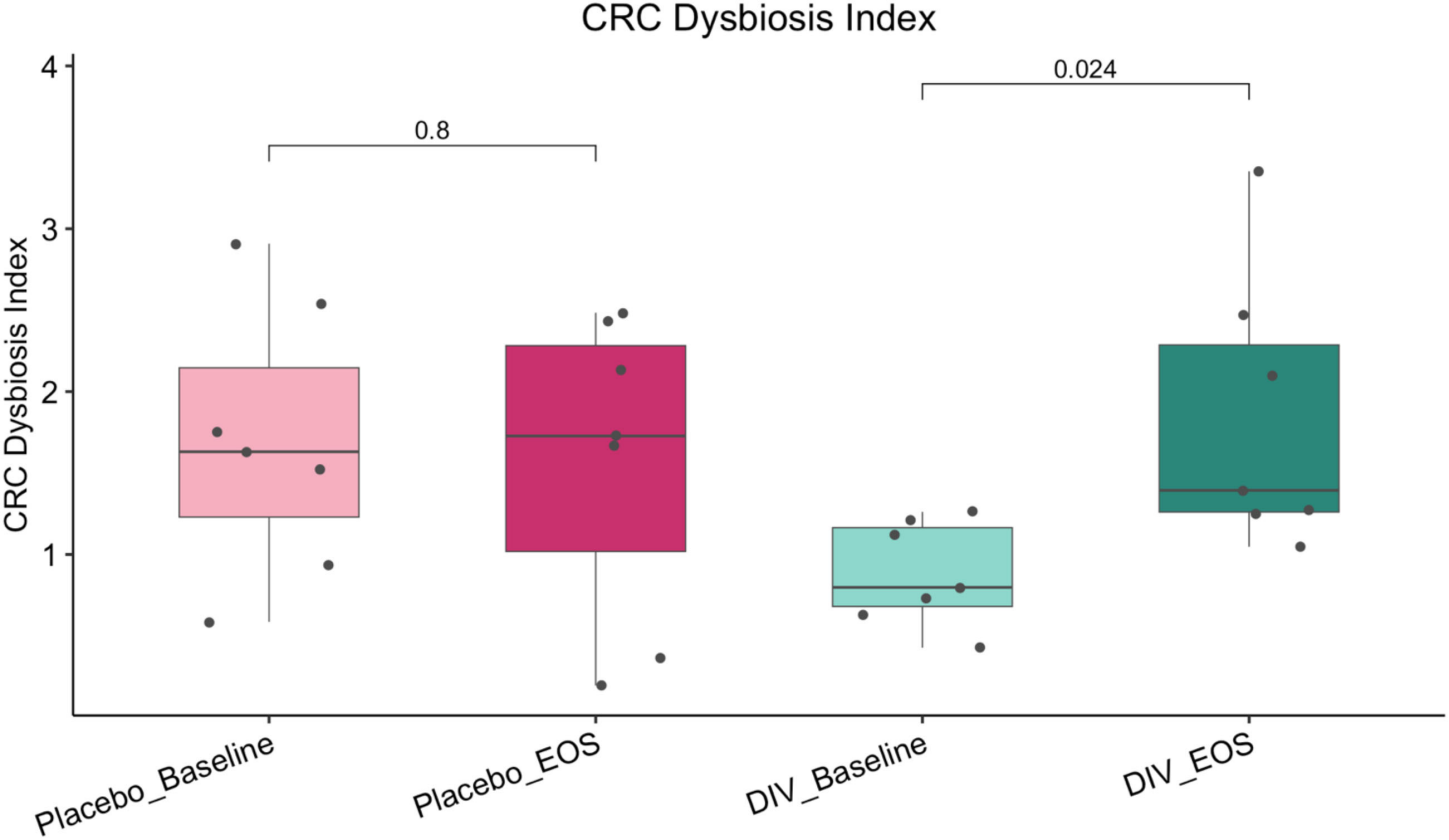
Effect of Del-Immune V on the colorectal cancer (CRC)-associated Dysbiosis Index (DI). Box-and-whisker plots depict the distribution of the DI at baseline and end of study (EOS) for participants receiving placebo (Placebo_Baseline and Placebo_EOS) or Del-Immune V (DIV_Baseline and DIV_EOS). Data are shown for the microbiome subset of 17 participants (placebo, n = 10; Del-Immune V, n = 7). The center line represents the median, boxes indicate the interquartile range (IQR), whiskers extend to 1.5 × IQR, and gray dots represent individual participant values. Within-group changes from baseline to EOS were assessed using the Wilcoxon signed-rank test. The Dysbiosis Index remained unchanged in the placebo group (*p* = 0.80), whereas the Del-Immune V group exhibited a significant change between baseline and EOS (*p* = 0.024), consistent with a shift toward a more favorable gut microbial profile.

The CRC dysbiosis index (DI) remained stable in the placebo arm from baseline to end-of-study (p = 0.8), whereas participants receiving Del-Immune V exhibited a significant increase in DI (p = 0.024; **Figure 4**), reflecting enrichment of protective taxa (*Lachnospiraceae*, *Bifidobacteriaceae*) relative to CRC-associated genera (*Fusobacterium, Alistipes, Parvimonas,Peptostreptococcus*).

#### 3.10.6 Predicted functional profiling of the del-immune V metagenome

Functional inference was performed using PICRUSt applied to 16S rRNA sequencing data. Intra-group changes within the Del-Immune V arm were assessed using Kruskal–Wallis testing, and between-group differences at end-of-study were identified using LEfSe. All nominal findings are reported; no feature survived FDR correction (minimum FDR-adjusted p = 0.73), consistent with the modest sample size and exploratory nature of these analyses.

Within the Del-Immune V arm, 15 species-level lineages showed nominal decreases in inferred abundance from baseline to end-of-study (p < 0.05). These included *Tissierellia*-associated taxa such as *Peptostreptococcus, Parvimonas*, and *Peptoniphilaceae* lineages, as well as the *Fusobacterium nucleatum* group, which declined from 0.47% to 0.002%. Several *Oscillospiraceae* members also decreased, including Faecalibacterium prausnitzii group (from approximately 5.9% to 2.2%), consistent with the overall contraction in alpha diversity following surgery and perioperative exposure. Among KEGG orthologs, intra-group analysis identified decreased predicted abundance of a lipooligosaccharide transport system ATP-binding protein (K09695) and an NADP-dependent formate dehydrogenase alpha subunit (K05299), while carbohydrate-utilization functions increased, including rhamnogalacturonan endolyase (K18197), alpha-glucuronidase (K01235), hexuronate and rhamnose transporters (K08191, K02856), and L-ribulose-5-phosphate 4-epimerase (K03077), collectively suggesting a shift toward enhanced utilization of complex plant polysaccharides.

**Figure 5:**
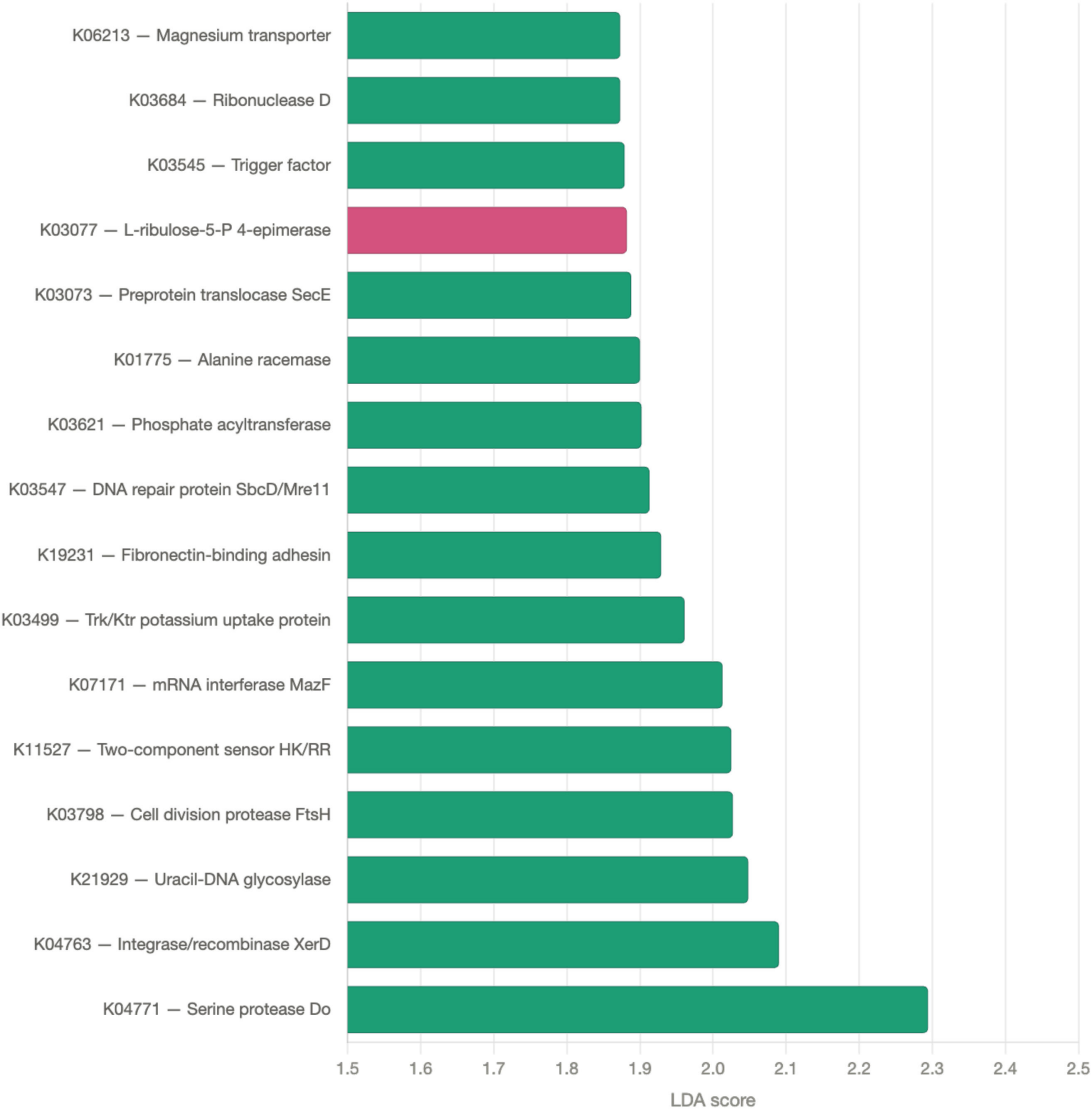
LEfSe analysis identifying differentially abundant KEGG orthologs between the Del-Immune V and placebo groups at the end of the study (EOS). Bars represent the linear discriminant analysis (LDA) score for each significantly discriminant KEGG ortholog. Green bars indicate functions enriched in the Del-Immune V group relative to placebo, whereas the pink bar indicates a function enriched in the placebo group relative to Del-Immune V. The x-axis shows the corresponding LDA score. Nominal p-values are shown; however, no feature remained significant after false discovery rate (FDR) correction (FDR-adjusted p ≥ 0.73).

Between-group LEfSe analysis at end-of-study identified 16 KEGG orthologs with LDA scores ≥ 2.0 that differed nominally between the Del-Immune V and placebo groups (**Figure 5**). Fifteen of the 16 discriminating orthologs were lower in the Del-Immune V arm relative to placebo at end-of-study, including serine protease Do (K04771; LDA = 2.29, p = 0.032), fibronectin-binding autotransporter adhesin (K19231; LDA = 1.93, p = 0.048), and mRNA interferase MazF (K07171; LDA = 2.01, p = 0.037) — functions associated with bacterial stress responses, pathogen-host adhesion, and toxin-antitoxin systems enriched in dysbiotic or virulence-associated communities. The single ortholog enriched in the Del-Immune V arm was L-ribulose-5-phosphate 4-epimerase (K03077; LDA = 1.88, p = 0.007), which channels arabinose and fucose into central carbon metabolism and is characteristic of saccharolytic fermenters such as Bifidobacterium and Lachnospiraceae. At the pathway level, the bacterial secretion system (ko03070; LDA = 2.68, p = 0.047) was decreased in the Del-Immune V group, while the D-glucuronate degradation module (M00061; LDA = 2.83, p = 0.043) was increased, consistent with enhanced complex carbohydrate fermentation capacity. The pyruvate oxidation module (M00307; LDA = 3.03, p = 0.044) was decreased in the Del-Immune V group, potentially reflecting reduced fermentative activity of anaerobic lineages associated with inflammatory or dysbiotic niches.

Collectively, these predicted functional shifts — toward saccharolytic carbohydrate metabolism and away from stress-, virulence-, and pathogen-associated functions — are directionally concordant with the taxonomic restructuring, dysbiosis index improvement, and selective IL-6 reduction observed in the Del-Immune V group, providing a hypothesis-generating functional framework consistent with the proposed microbiome–immune axis.

## 4 Discussion

The comparability of baseline characteristics between study groups is a critical prerequisite for ensuring the validity of outcome analyses. In this study, both groups were largely similar across demographic and clinical variables, including age, sex, body mass index (BMI), comorbidities, preoperative biomarker levels, and tumor characteristics. This balance supports the reliability of subsequent comparisons.

However, the higher proportion of Stage IV disease in the Del-Immune V group (22.7% vs. 0%, p = 0.056) represents a notable imbalance that may act as a confounding factor. The inclusion of patients with more advanced disease in the intervention arm could attenuate the apparent beneficial effects of the metabiotic, thereby underestimating its potential impact. This limitation underscores the importance of interpreting outcomes with caution and highlights the need for subgroup analyses stratified by tumor stage in future studies. Such stratification would allow for a more nuanced understanding of whether Del-Immune V exerts differential effects depending on disease severity (Hernandez Dominguez et al., 2023; Sherry et al., 2023).

The stability of BMI across both groups during the 8-week follow-up is consistent with the principles of the Enhanced Recovery After Surgery (ERAS) protocol for colorectal cancer surgery (Gustafsson et al., 2025). ERAS emphasizes early mobilization and nutritional support, which likely contributed to the preservation of body composition in this cohort. Nevertheless, BMI is a crude measure that does not capture muscle mass or sarcopenia (Marshall and White, 2019), both of which are more sensitive indicators of nutritional decline and may warrant further investigation in future studies.

Previous reports have shown that application of the ERAS protocol to colorectal laparoscopic surgery attenuates the surgical stress response, leading to postoperative reductions in IL-6 and CRP levels (Mari et al., 2016). This effect was further enhanced in our study, where IL-6 decreased significantly only in the Del-Immune V group (p = 0.012), suggesting a specific immunomodulatory role of the intervention. Compared with other bacterial lysates such as liastenum (*Lactobacillus delbrueckii*), deodan (*Lactobacillus bulgaricus*), or BCG derivatives, whose immunomodulatory activity is primarily mediated through muramyl dipeptides and peptidoglycans, Del−Immune V offers distinctive advantages. It combines peptidoglycan fragments with bacterial DNA from *Lactobacillus rhamnosus* V, enabling dual activation of Toll−like receptors and NOD−like receptors, thereby inducing a broader cytokine response (IL−1, IL−6, TNF−α, IFN−γ) and enhancing NK cell activity (Sichel et al., 2013). Importantly, Del−Immune V is derived from a GRAS strain and registered as a food supplement with the FDA, ensuring a strong safety profile and clinical feasibility. Preclinical studies have demonstrated dose−dependent interferonogenic activity, supporting its translation into human trials. In the specific context of colorectal cancer, where perioperative immune competence and modulation of systemic inflammation are critical, Del−Immune V’s dual mechanism and regulatory approval make it a more suitable candidate than other lysates for clinical evaluation (Sichel et al., 2013).

Given that IL-6 is a central mediator of systemic inflammation and has been associated with poor prognosis in colorectal cancer (Turano et al., 2021), this reduction may represent a clinically meaningful benefit (Feng et al., 2023). CRP levels decreased significantly in both groups (p < 0.001 and p = 0.001), reflecting the expected resolution of postoperative inflammation. However, the trend toward greater reduction in the intervention group suggests that Del-Immune V may augment the natural recovery process (Meng et al., 2021; Gwenzi et al., 2024).Similarly, the observed trend toward reduced CEA levels in the Del-Immune V group (p = 0.076) could indicate a potential effect on residual tumor burden or inflammatory activity, though this requires confirmation in larger cohorts (Titu et al., 2025) Importantly, the consistent directionality of effects—greater reduction and normalization in the Del-Immune V group across all biomarkers—suggests a potential biological effect of the metabiotic that may achieve statistical significance in larger studies. Considerable interindividual variability was observed in both groups, as reflected by wide interquartile ranges, underscoring the heterogeneity of inflammatory and tumor responses in colorectal cancer patients. Taken together, these findings support the hypothesis that Del-Immune V modulates the perioperative inflammatory response, particularly through reduction of IL-6, and highlight the need for larger studies to confirm these effects with greater statistical precision.

From a mechanistic standpoint, the microbiome findings align with the pattern of inflammatory biomarker changes observed in this study. Alpha-diversity metrics showed that both groups experienced a contraction in richness and diversity between baseline and end-of-study (e.g., Chao1 decreased from 837.25 ± 137.4 to 694.16 ± 121.5 in the Del-Immune V group and from 668.94 ± 109.8 to 648.47 ± 92.6 in the placebo group), consistent with the impact of colorectal surgery, perioperative antibiotics, and diet on the gut ecosystem. Within this globally constrained diversity space, however, Del-Immune V directed the microbiome toward a more anti-inflammatory configuration: SCFA-producing commensals such as *Bifidobacterium, Agathobacter*, and *Gemmiger* were enriched, primary carbohydrate degraders including *Segatella* and *Phocaeicola* increased, and the CRC-associated taxon *Fusobacterium* declined relative to both baseline and placebo at end-of-study. When these protective and oncogenic taxa were integrated into a composite CRC dysbiosis index, only the Del-Immune V group demonstrated a significant increase from baseline to end-of-study (p = 0.024), indicating a net shift away from a *Fusobacterium/Alistipes/Peptostreptococcus*-enriched profile toward one dominated by *Lachnospiraceae*- and *Bifidobacteriaceae*-associated guilds.

This ecological trajectory mirrors the clinical biomarker profile. The intervention arm exhibited a larger median percentage reduction in IL-6 (−45.2% vs. −28.7%) and CRP (−62.8% vs. −48.3%) and higher proportions of patients achieving IL-6 and CRP normalization (63.6% vs. 47.1% and 81.8% vs. 70.6%, respectively), albeit without statistically significant between-group differences. Fusobacterium-rich dysbiosis has been linked to activation of TLR4/MYD88/NF-κB and amplification of IL−6–driven inflammatory signaling in colorectal cancer, whereas butyrate- and acetate-producing commensals from the Lachnospiraceae and Bifidobacteriaceae families are associated with reduced mucosal inflammation and improved barrier integrity. The enrichment of SCFA-producing guilds together with a decline in Fusobacterium under Del-Immune V provides a biologically plausible framework consistent with the observed reduction in IL-6; however, causality cannot be established in the present study, and larger studies incorporating microbiome-metabolome-host biomarker integration will be required to formally test these relationships.

While the present trial was not powered to detect robust correlations between individual taxa or dysbiosis index values and specific biomarkers, the concordant direction of microbiome restructuring and inflammatory marker changes supports the hypothesis that Del-Immune V modulates the perioperative inflammatory response, at least in part, through remodeling of the gut microbial ecosystem (Sánchez-Alcoholado et al., 2020; Artemev et al., 2022; Thulasinathan et al., 2025).

The analysis of Advance and beneficial effects carry several important clinical implications. Del-Immune V was safe and well tolerated, with an adverse effect profile comparable to placebo. Mild gastrointestinal symptoms, which are expected with metabiotics, did not necessitate treatment discontinuation, underscoring the product’s favorable tolerability. The potential effect on anxiety observed in some patients warrants further investigation in studies specifically designed to evaluate mental health outcomes (Renna et al., 2022). Some trials employed probiotics reported improvements in anxiety, and depressive symptoms, while others showed no significant differences compared with control groups (Amirkhanzadeh Barandouzi et al., 2026). Importantly, the absence of serious adverse events supports the continued clinical development of Del-Immune V, provided that continuous safety monitoring is maintained in subsequent phases of evaluation.

Analysis of colorectal cancer–specific symptoms revealed significant intra-group improvements in the Del-Immune V arm, with 7 of 12 scales showing statistically significant changes (p < 0.05). In contrast, the placebo group demonstrated no significant intra-group improvements, although some positive trends were noted. Direct inter-group comparisons at endpoint did not reach statistical significance (all p > 0.05), likely reflecting the limited sample size.

Nevertheless, the magnitude of change consistently favored Del-Immune V, with greater improvements in functional scales and symptom reductions. Global quality of life improved by +8.4 points in the Del-Immune V group compared to +3.0 points in placebo, yielding a 5.4-point difference that exceeds the threshold for clinically meaningful change (5–10 points according to EORTC literature (Musoro et al., 2023). Symptom-specific improvements, including reductions in abdominal pain and blood in stools, align with the proposed intestinal health mechanism of the metabiotic.

These findings suggest that Del-Immune V may enhance postoperative functional recovery in colorectal cancer patients, particularly in physical, emotional, and social domains. Symptom reduction may also contribute to better adherence to adjuvant therapy, thereby supporting long-term treatment outcomes. Future studies with larger sample sizes and extended follow-up (6–12 months) are warranted to confirm these preliminary observations and to explore the durability of quality-of-life benefits.

Microbiome analysis revealed that administration of Del−Immune V was associated with measurable restructuring of the intestinal ecosystem. Specifically, patients in the intervention group demonstrated enrichment of beneficial fermentative taxa, alongside reductions in microbial groups commonly linked to colorectal cancer–related dysbiosis. Collectively, these findings suggest that Del−Immune V influences the gut ecosystem through immune–microbial ecological modulation rather than direct colonization, promoting a microbial configuration associated with enhanced short−chain fatty acid (SCFA) production and improved intestinal metabolic balance (Speckmann et al., 2024). Administration of Del−Immune V, a metabiotic derived from enzymatically digested *Lacticaseibacillus rhamnosus*, is hypothesized to modulate mucosal immune signaling and alter microbial ecological niches within the intestinal environment (Sharma and Shukla, 2020). These changes favor expansion of fermentative taxa such as Bifidobacterium and members of the Lachnospiraceae family, resulting in increased SCFA production and enhanced epithelial barrier support (Parada Venegas et al., 2019; Fusco et al., 2023). Conversely, the resulting microbial environment suppresses the expansion of colorectal cancer–associated anaerobic taxa including *Fusobacterium, Parvimonas*, and *Peptostreptococcus*, thereby contributing to improved microbial ecosystem balance (Modeel et al., 2026; Yang et al., 2026).

Functional predictions derived from PICRUSt analysis revealed that Del−Immune V administration was associated with increased microbial capacity for complex carbohydrate utilization and polysaccharide degradation (Yin and Wang, 2021). Enzymes involved in plant fiber breakdown and carbohydrate metabolism, including beta−glucosidase, xylanases, arabinan−degrading enzymes, and SusD/TonB transport systems (Sheridan et al., 2016) showed increased abundance in end−of−study samples. These functional changes are consistent with the taxonomic enrichment of fermentative taxa such as Bifidobacterium and members of the *Lachnospiraceae* family, which are known to participate in dietary carbohydrate breakdown and short−chain fatty acid (SCFA) production (Maukonen and Ouwehand, 2022; Fusco et al., 2023; Fitzgerald et al., 2025).

Enhanced capacity for saccharolytic fermentation supports cross−feeding metabolic networks that generate acetate and butyrate, metabolites with key roles in epithelial barrier maintenance and anti−inflammatory immune regulation (Siddiqui and Cresci, 2021). In parallel, several functions related to stress response, DNA replication, and general metabolic maintenance decreased following treatment, a pattern that may reflect reduced ecological pressure associated with inflammatory or opportunistic niches in the gut environment (Shen et al., 2025).

Taken together, the PICRUSt analysis suggests that Del−Immune V promotes a functional shift toward fiber utilization and SCFA production, consistent with the taxonomic restructuring observed in the microbiome data). This functional reorientation provides a mechanistic explanation for the observed improvements in inflammatory biomarkers and colorectal cancer–specific symptoms, reinforcing the potential of metabiotic to modulate host–microbiome interactions in the perioperative setting.

### 4.1 Microbiome analysis

Consistent with the alpha diversity profiles shown in **Figure 2**, Del-Immune V produced measurable but moderate changes in within-sample diversity, characterized by reduced estimated richness and increased community evenness over the intervention period.

Specifically, Chao-estimated richness decreased in both Del-Immune V and placebo groups, with a more pronounced reduction in the intervention arm, while Shannon diversity showed a modest decline and Simpson diversity increased, particularly among participants receiving Del-Immune V (**Figure 2**). Alpha diversity indices such as Chao, Shannon, and Simpson (**Table 9**) capture complementary aspects of richness and evenness within a single community and are widely used to summarize gut microbiome structure in clinical and interventional studies. In this context, the concurrent reduction in richness and increase in Simpson diversity observed in **Figure 2** suggests a consolidation of the microbial community, with depletion of some low-abundance taxa and a more even distribution among the remaining taxa, rather than a simple loss of overall diversity (Perzon and Ilan, 2025; Éliás et al., 2026).

As reported in prior probiotic and synbiotic trials, conventional alpha diversity metrics in generally healthy or mildly symptomatic populations often show small or negligible changes, even when taxonomic or functional shifts are present. A recent synthesis of probiotic intervention studies in adults noted that indices such as Shannon, Chao1, and Simpson frequently exhibit limited responsiveness, underscoring that community-level diversity measures may be relatively insensitive to targeted microbial modulation. Our findings, as summarized in **Figure 1**, are congruent with this literature: Del-Immune V did not induce a dramatic increase in richness or Shannon diversity but did alter the balance of taxa sufficiently to increase Simpson diversity, consistent with a more even distribution of taxa within the community. Such changes in evenness may reflect selective modulation of specific bacterial groups without wholesale restructuring of the entire microbiota (Wang et al., 2022; Rodenes-Gavidia et al., 2023; Perzon and Ilan, 2025; Éliás et al., 2026).

From a biological standpoint, greater microbial evenness has been proposed as a feature of more resilient ecosystems, as communities with reduced dominance by a small number of taxa may display enhanced functional redundancy and stability. However, the health implications of modest shifts in richness and evenness are context-dependent and influenced by which taxa are lost or gained, as well as their metabolic capacities and interactions with the host. In our study, the decrease in richness and Shannon diversity combined with an increase in Simpson diversity in the Del-Immune V group (**Figure 2**) suggests a more organized community structure but does not, on its own, establish whether these changes are beneficial, neutral, or unfavorable with respect to long-term colorectal cancer risk. Accordingly, we interpret the alpha diversity findings illustrated in **Figure 2** as evidence of subtle remodeling of gut microbial community structure, which should be integrated with taxon-level analyses and dysbiosis index data when evaluating the overall microbiome impact of Del-Immune V (Yang et al., 2021; Feizi et al., 2022; Kvakova et al., 2022; Cui, 2025; Kang et al., 2025; Éliás et al., 2026).

#### 4.1.1 Functional guild restructuring under del-immune V

Beyond individual taxon shifts, functional guild analysis revealed a coordinated reorganization of the gut microbial community in the Del-Immune V arm that was not observed in the placebo group (**Table 11**). The most pronounced changes occurred within two guilds: the SCFA-producing commensals and the opportunistic/dysbiosis-associated taxa. By end-of-study, participants receiving Del-Immune V showed enrichment of Bifidobacterium (+1.94 percentage points vs. placebo), *Agathobacter*, and *Gemmiger* — all acetate- and butyrate-producing genera whose metabolites serve as the preferred energy substrate for colonocytes and reinforce epithelial barrier function (**Table 12**). Concurrently, the CRC-associated taxon *Fusobacterium* declined in the Del-Immune V arm while persisting at 2.04% in the placebo microbiome at end-of-study (**Table 12**), suggesting that the intervention selectively disfavored facultative anaerobes linked to chronic inflammation and tumorigenesis.

Among primary carbohydrate fermenters, *Segatella* exhibited the largest differential enrichment between arms (+7.38; **Table 12**), accompanied by a moderate increase in *Phocaeicola* (+1.45). The expansion of these polysaccharide degraders may reflect enhanced substrate availability for downstream cross-feeding, as lactic acid producers (*Ligilactobacillus, Streptococcus*) also showed slight enrichment in the Del-Immune V arm (**Table 11**). This pattern is consistent with a syntrophic cascade in which primary degraders liberate oligosaccharides and lactate that are subsequently metabolized by SCFA-producing commensals and secondary fermenters — a trophic network that has been proposed as a hallmark of a resilient, health-associated gut ecosystem (Safarchi et al., 2025).

Two observations require cautious interpretation. First, Akkermansia, a mucin-degrading specialist associated with metabolic health, declined in the Del-Immune V cohort (**Table 11**). While this could reflect competition for mucosal niche space as other commensals expanded, it may also indicate transient remodeling of the mucus layer under immune stimulation. Second, Escherichia was elevated in the Del-Immune V arm (+3.01 vs. placebo; **Table 12**), an increase that was classified as opportunistic.

#### 4.1.2 Dysbiosis index

To formalize the balance between these protective and oncogenic guilds into a single testable metric, we calculated a CRC dysbiosis index (DI) defined as the log-ratio of *Lachnospiraceae* plus *Bifidobacteriaceae* to *Fusobacterium, Alistipes*, and *Peptostreptococcus*, such that higher values indicate a microbiome configuration shifted away from CRC-associated dysbiosis. The DI remained stable in the placebo arm across timepoints (p = 0.8), whereas participants receiving Del-Immune V exhibited a significant increase in DI from baseline to end-of-study (p = 0.024; **Figure 4**). This increase is concordant with the guild-level findings: enrichment of SCFA-producing *Bifidobacterium* and *Lachnospiraceae* members (*Agathobacter, Gemmiger, Blautia*) in the numerator, coupled with a decline in *Fusobacterium* in the denominator, drove the index upward toward a more protective profile (**Tables 11**, **12**).

Each denominator taxon contributes to colorectal tumorigenesis through distinct but convergent pro-inflammatory mechanisms. *Fusobacterium nucleatum* activates the TLR4/MYD88/NF−κB signaling cascade, enhances oncogenic Wnt/β−catenin signaling, and suppresses NK−cell and CD8^+^ T−cell cytotoxicity via the Fap2–TIGIT and CEACAM1 inhibitory axes, collectively fostering an immunosuppressive tumor microenvironment that promotes CRC progression and chemoresistance (Gur et al., 2015; Chen et al., 2017; Dadgar-Zankbar et al., 2024).

*Alistipes* is enriched in CRC tissue relative to adenomas and is associated with a pro−inflammatory milieu characterized by elevated TNF−α and IL−6, which may activate the LRG1/TGF−β1 angiogenic signaling pathway implicated in early CRC carcinogenesis (Fu et al., 2024; Rathore et al., 2025).

*Peptostreptococcus anaerobius* engages integrin α2β1 on CRC cells to activate NF-κB/CXCL1 signaling, recruiting myeloid-derived suppressor cells (MDSCs) into the tumor microenvironment and thereby suppressing T-cell-mediated antitumor immunity and driving resistance to anti-PD1 immunotherapy (Dadgar-Zankbar et al., 2024; Fu et al., 2024; Li et al., 2024; Liu et al., 2024; Mondal et al., 2025). In this context, the dose-response data strongly support the interpretation that Del-Immune V is not merely “stimulating immunity,” but may be inducing a temporary shift in immune system organization (Kang et al., 2018). The evidence that approximately 50 µg is the optimal dose, with diminishing returns at higher doses, suggests nonlinear interaction behavior characteristic of complex biological processes (Beltrán-Velasco and Clemente-Suárez, 2025). This aligns with the transient interferon response and the need for repeated dosing, indicating that the product may be restoring coherence in immune signaling environments (Sichel et al., 2013).

Del-Immune V is a metabiotic derived from *Lactobacillus rhamnosus* DV cell wall fragments that engage pattern-recognition receptors — particularly TLR2 — on intestinal epithelial cells and dendritic cells, modulating both pro- and anti-inflammatory cytokine cascades. This immunomodulatory stimulus may create luminal conditions that favor the expansion of SCFA-producing commensals at the expense of inflammation-associated genera, as reflected in both the guild restructuring (**Table 11**) and the significant increase in the DI (Plantinga et al., 2011).

In the present study, the DI remained stable in the placebo arm across timepoints (p = 0.8), whereas participants receiving Del-Immune V exhibited a significant increase in DI from baseline to end-of-study (p = 0.024; **Figure 4**). This increase reflects a measurable shift away from a CRC-associated profile, driven by the enrichment of *Bifidobacterium, Agathobacter*, and *Gemmiger* and a concurrent decline in *Fusobacterium* in the Del-Immune V cohort (**Table 10**). Del-Immune V is a metabiotic derived from *Lactobacillus rhamnosus* DV cell wall fragments that engage pattern-recognition receptors, particularly TLR2, on intestinal epithelial cells and dendritic cells, modulating both pro- and anti-inflammatory cytokine cascades (TNF-α, IL-6, IL-10, IL-12). This immunomodulatory stimulus may create ecological conditions in the gut lumen that favor the expansion of SCFA-producing commensals at the expense of opportunistic, inflammation-associated genera, as reflected by the functional guild analysis (**Table 11**) (Plantinga et al., 2011; von Ossowski et al., 2013).

The DI integrates taxa across two taxonomic levels (family and genus), capturing broad ecological shifts rather than single-species perturbations, an approach consistent with emerging consensus that disease-associated dysbiosis is best characterized by community-level imbalances among functional guilds rather than the presence or absence of individual pathogens. However, the index does not account for strain-level variation, nor does it incorporate functional metagenomic data such as butyrate biosynthesis gene abundance or fecal short-chain fatty acid concentrations. Future studies should pair the DI with targeted metabolomic profiling to determine whether the taxonomic restructuring observed here translates to measurable changes in the colonic metabolome and immune signaling landscape (Wei et al., 2021; Almonte et al., 2026).

#### 4.1.3 Integration of microbiome and clinical responses

Alpha-diversity metrics provide important context for interpreting the guild-level restructuring observed under Del-Immune V. At baseline, the Del-Immune V cohort exhibited higher estimated richness (Chao1 837.25 ± 137.4 vs. 668.94 ± 109.8) and greater Shannon diversity (4.05 ± 0.39 vs. 3.67 ± 0.35) than the placebo group. By end-of-study, both arms showed numerical decreases in richness and diversity, consistent with the impact of surgery, perioperative antibiotics, and dietary perturbations on the gut microbiome (DIV_EOS Chao1 694.16 ± 121.5, Shannon 3.63 ± 0.33; Placebo_EOS Chao1 648.47 ± 92.6, Shannon 3.59 ± 0.31). These parallel declines suggest that Del-Immune V did not prevent the global contraction in alpha diversity associated with colorectal cancer surgery and recovery, but rather reshaped community composition within this constrained diversity space.

Within that context, the functional guild and dysbiosis index results indicate that Del-Immune V redirected the recovering microbiome toward a more anti-inflammatory configuration. The intervention arm demonstrated enrichment of SCFA-producing commensals (*Bifidobacterium, Agathobacter, Gemmiger*) and primary carbohydrate degraders (*Segatella, Phocaeicola*), alongside a decline in the CRC-associated taxon *Fusobacterium*, while the placebo microbiome retained a measurable Fusobacterium signal at end-of-study. When these protective and oncogenic taxa were integrated into the CRC dysbiosis index, only the Del-Immune V group showed a significant increase in DI from baseline to end-of-study (p = 0.024), consistent with a shift away from a *Fusobacterium/Alistipes/Peptostreptococcus*-enriched profile toward one dominated by *Lachnospiraceae*- and *Bifidobacteriaceae*-associated functional guilds.

This microbiome trajectory parallels the clinical biomarker pattern. IL-6, a central mediator of systemic inflammation and poor colorectal cancer prognosis (Guthrie et al., 2013; Xu et al., 2016), decreased significantly only in the Del-Immune V group (p = 0.012), whereas CRP declined in both groups with a trend toward greater normalization under Del-Immune V.

*Fusobacterium*-rich dysbiosis has been linked to activation of TLR4/MYD88/NF-κB and amplification of IL-6–driven inflammatory signaling in CRC, while butyrate- and acetate-producing commensals such as Lachnospiraceae and Bifidobacterium have been associated with reduced mucosal inflammation and improved barrier integrity (Yang et al., 2017; Ou et al., 2022).

The observed enrichment of SCFA-producing guilds together with a decline in *Fusobacterium* under Del-Immune V therefore provides a plausible ecological mechanism for the selective reduction in IL-6 and the more favorable trajectory of CRP and CEA in the intervention arm. Although this study was not powered to detect correlations between individual taxa or DI values and specific biomarkers, the concordant direction of microbiome restructuring and inflammatory marker changes supports the hypothesis that Del-Immune V modulates the perioperative inflammatory response in part through remodeling of the gut microbial ecosystem (Sánchez-Alcoholado et al., 2020; Artemev et al., 2022; Thulasinathan et al., 2025).

These findings should be interpreted with caution given the modest sample size, baseline differences in disease stage, and lack of strain-level or metagenomic functional data. Nonetheless, the combination of (i) preserved but slightly contracted alpha diversity in both arms, (ii) guild-level shifts toward SCFA-producing commensals and away from CRC-associated taxa uniquely in the Del-Immune V group, and (iii) preferential reductions in IL-6 and trends toward greater CRP and CEA normalization in the same group, suggests a coherent microbiome–immune axis that warrants confirmation in larger, mechanistically focused trials.

#### 4.1.4 interpreting PICRUSt predictions

The PICRUSt-derived functional signatures provide additional, hypothesis-generating support for the taxonomic and clinical patterns observed in this trial. At the taxon level, the nominally significant decreases in *Tissierellia*-associated genera (Peptostreptococcus*, Parvimonas, Peptoniphilaceae*) and in the *Fusobacterium nucleatum* group are consistent with the observed decline in CRC- and inflammation-associated taxa in the Del-Immune V arm, as captured by both the guild analysis and the CRC dysbiosis index. These lineages have been linked to pro-inflammatory signaling, impaired barrier function, and adverse oncologic outcomes in colorectal cancer, and their reduction over the 8-week intervention period aligns with the selective, statistically significant decrease in IL-6 and the numerically greater improvements in CRP and CEA in the Del-Immune V group. At the same time, the decline in beneficial *Oscillospiraceae* such as *Faecalibacterium prausnitzii* underscores the impact of surgery and perioperative stress on the microbiome and highlights that Del-Immune V appears to redirect a generally contracted community rather than fully preserving canonical “health-associated” taxa.

Functionally, the predicted reduction in lipooligosaccharide transport capacity (K09695) and formate dehydrogenase activity (K05299) may reflect a shift away from Gram-negative–associated endotoxin handling and specific anaerobic respiratory pathways that can sustain inflammation in the post-operative colon. In parallel, the increased inferred abundance of enzymes and transporters involved in the degradation and uptake of rhamnogalacturonan, hexuronates, and rhamnose (K18197, K01235, K08191, K02856, K03077) is concordant with the observed enrichment of primary carbohydrate degraders and SCFA-producing commensals in the Del-Immune V arm, suggesting an enhanced capacity to harvest and ferment complex dietary polysaccharides into short-chain fatty acids. Given that butyrate and related SCFAs have been shown to dampen mucosal inflammation, support epithelial repair, and modulate antitumor immunity, these predicted functional changes offer a plausible mechanistic bridge between the ecological restructuring of the microbiome and the observed improvements in systemic inflammatory markers (Sánchez-Alcoholado et al., 2020; Thulasinathan et al., 2025).

However, these PICRUSt findings must be interpreted with caution. No taxon or ortholog remained significant after FDR correction, the predictions are inherently constrained by 16S rRNA gene-based reference genomes, and the modest sample size limits power to detect subtle functional shifts. Moreover, the mixed behavior of certain commensal lineages (e.g., reductions in Faecalibacterium prausnitzii despite an overall shift toward SCFA-producing guilds) illustrates the complexity of microbiome recovery after colorectal surgery. Future studies should complement PICRUSt-based inference with shotgun metagenomics and targeted metabolomics to directly quantify SCFA production, lipopolysaccharide/lipooligosaccharide biosynthesis capacity, and other pathways suggested here, and to formally test correlations between functional profiles, the CRC dysbiosis index, and perioperative biomarkers such as IL-6, CRP, and CEA.

The between-group LEfSe analysis at end-of-study provides additional functional support for the ecological restructuring observed in the Del-Immune V arm (**Figure 5**). The predominant finding — a broad reduction in predicted stress response, virulence, and pathogen-associated functions — is mechanistically coherent with the decline in CRC-associated taxa documented in the taxonomic and dysbiosis index analyses. Specifically, the decreased predicted abundance of fibronectin-binding adhesin (K19231), mRNA interferase *MazF* (K07171), and bacterial secretion system capacity (ko03070) in the Del-Immune V microbiome suggests a community less enriched in organisms relying on host adhesion, stress survival, and type II/III secretion-dependent virulence — functional hallmarks of the dysbiotic, inflammation-promoting state associated with CRC progression. Conversely, the enrichment of L-ribulose-5-phosphate 4-epimerase (K03077) and D-glucuronate degradation capacity (M00061) in the Del-Immune V arm provides functional corroboration of the taxonomic expansion of saccharolytic fermenters such as *Segatella (*Panwar et al., 2025)*, Phocaeicola* (Lück and Deppenmeier, 2022)*, and Bifidobacterium* (Fusco et al., 2023), whose growth depends on these enzymatic pathways for complex carbohydrate breakdown and short-chain fatty acid production. Taken together, the LEfSe functional profile and the taxonomic guild analysis tell a consistent story: Del-Immune V redirected the post-surgical microbiome toward a metabolic configuration associated with epithelial support and mucosal homeostasis, and away from one associated with bacterial stress, inflammation, and oncogenic signaling. While causality cannot be established from these data and no feature survived FDR correction, the directional concordance across taxonomic, functional, and clinical domains strengthens the overall mechanistic narrative and justifies further investigation in larger, mechanistically powered trials.

#### 4.1.5 Conclusion, limitations and future directions

Del-Immune V demonstrated perioperative immunomodulatory activity in colorectal cancer patients, consistent with the objectives of Enhanced Recovery After Surgery (ERAS) protocols. The intervention was associated with reductions in IL-6 and CRP, improvements in patient-reported quality of life, and restructuring of the gut microbiome characterized by enrichment of protective commensals and suppression of CRC-associated taxa. These author findings suggest that Del-Immune V may serve as a safe and clinically meaningful adjunct to ERAS-guided recovery. However, we acknowledge that the study duration and follow-up do not allow definitive conclusions regarding long-term oncologic outcomes. Extended surgical endpoints are being addressed in a separate publication derived from the same clinical program. Accordingly, the present manuscript focuses on perioperative host–microbiome interactions and biomarker dynamics, while future multicenter studies with longer follow-up will be essential to determine the durability and translational relevance of these effects.

This study provides preliminary evidence that Del-Immune V modulates the perioperative inflammatory response in colorectal cancer patients through restructuring of the gut microbiome. However, several limitations must be acknowledged. First, the modest sample size (n=39), while calculated to provide 80% power to detect a 40% reduction in IL-6 based on prior pilot data, reduces statistical power for secondary endpoints and increases susceptibility to baseline imbalances, such as the higher proportion of Stage IV disease in the intervention arm. Consequently, while the directionality of the microbiome and clinical findings is consistent and statistically significant in key areas (e.g., IL-6 reduction, dysbiosis index), these exploratory results must be interpreted with caution and confirmed in larger, multicenter Phase II/III trials.

The reliance on 16S rRNA sequencing constrains taxonomic resolution and functional inference, preventing strain−level characterization and direct measurement of metabolic pathways. In addition, perioperative factors—including surgery, antibiotics, and diet—exerted strong ecological pressures that complicate attribution of changes solely to the intervention. The observed variability in commensal lineages, such as reductions in Faecalibacterium prausnitzii despite enrichment of SCFA−producing guilds, underscores the complexity of microbiome recovery in this clinical context.

Despite these limitations, the consistent directionality of findings across ecological, functional, and clinical domains is encouraging. Del−Immune V was associated with subtle but coherent shifts in alpha diversity, enrichment of fermentative taxa, suppression of CRC−associated lineages such as Fusobacterium, and increased dysbiosis index values. These ecological changes were accompanied by selective reductions in IL−6, favorable trends in CRP and CEA, and improvements in patient−reported outcomes, all within a favorable safety profile. Taken together, these results support the hypothesis of a microbiome–immune axis through which metabiotics may influence perioperative recovery and long−term oncologic trajectories.

Future studies should build on these observations with larger, multicenter cohorts and extended follow−up to assess durability of effects. Shotgun metagenomics and targeted metabolomics are needed to validate functional predictions, quantify SCFA production, and explore pathways such as lipopolysaccharide biosynthesis and host–microbe immune signaling. Stratified analyses by tumor stage and treatment modality will clarify whether benefits vary with disease severity. Finally, integration of microbiome data with clinical endpoints—including adherence to adjuvant therapy, recurrence rates, and survival—will be essential to determine the translational relevance of Del−Immune V in colorectal cancer care.

## Data Availability

The microbiome sequencing data generated and analyzed in this study are proprietary to Stellar Biotics and are not publicly available. Requests for access to the raw datasets should be directed to Dr. Libov Sichel at Stellar Biotics and will be considered on a case-by-case basis under appropriate data use agreements. Sequence read data will be deposited in the NCBI Sequence Read Archive (SRA), and accession numbers will be provided upon acceptance and publication of the article.
